# Factors influencing patients’ willingness to share their digital health data for primary and secondary use: A theory- and evidence-based overview of reviews

**DOI:** 10.1177/20552076251340254

**Published:** 2025-06-30

**Authors:** Sabrina Fesl, Caroline Lang, Jochen Schmitt, Stefanie Brückner, Stephen Gilbert, Stefanie Deckert, Madlen Scheibe

**Affiliations:** 1 Center for Evidence-Based Healthcare, Medical Faculty and University Hospital Carl Gustav Carus, TUD Dresden University of Technology, Dresden, Saxony, Germany; 2 Else Kröner Fresenius Center for Digital Health, TUD Dresden University of Technology, Dresden, Saxony, Germany

**Keywords:** electronic health records, personal health records, patient portals, health data, medical records, health information exchange, patient participation, informed consent, medical informatics, technology acceptance model

## Abstract

**Background:**

The sharing of health data (HD) remains intensely debated, such as in the context of the European Health Data Space. While HD sharing has great potential, the factors influencing patients’ willingness to share their HD remain unclear. Understanding patients’ perspectives is crucial to enhancing their motivation to share their HD, both with healthcare providers (primary use [PU]) and for purposes unrelated to patient care (secondary use [SU]).

**Objective:**

This overview of systematic reviews (SRs) synthesizes and qualitatively appraises available research on factors affecting patients’ willingness to share their digital HD for PU and SU.

**Methods:**

The MEDLINE, Embase, and Scopus literature databases were searched in June 2023, complemented by additional searches, to identify SRs focusing on the determinants of patients’ willingness to share HD published from 2013 to 2023. SRs underwent a multistage screening process using the inclusion and exclusion criteria based on the Population, Concept/Construct, and Context (PCC) framework, followed by data extraction and quality assessment using revised measurement tool to assess systematic reviews (R-AMSTAR2). Factors were categorized through a combined deductive-inductive thematic coding process, considering theories on HD sharing and technology acceptance.

**Results:**

Eleven SRs published between 2014 and 2021 were included, encompassing 321 articles with diverse study designs. Forty-one influencing factors (labeled as facilitators, barriers and inconsistent for unclassifiable factors) were identified and grouped into 15 main categories: 22 facilitators, nine barriers, and four inconsistent factors for PU and 13 facilitators, six barriers, and six inconsistent factors for SU. The key facilitators for PU and SU included higher education, trust, confidentiality, and transparency. The major barriers for PU and SU were privacy and security concerns. The R-AMSTAR2 overall confidence rating of all SRs was critically low.

**Conclusion:**

Our findings highlight modifiable and nonmodifiable factors affecting patients’ willingness to share their HD. Policymakers and healthcare providers should focus on modifiable factors such as individual usefulness, public benefit, and privacy and security concerns. High-quality SRs are urgently needed to provide reliable recommendations and to develop a holistic, practical framework.

**Protocol registration number:**

CRD42023429302

## Introduction

### Background

Large amounts of various types of health data (HD) are generated worldwide, especially in medical healthcare via regular treatment by healthcare providers. According to the EU General Data Protection Regulation (GDPR), HD is defined as all data pertaining to the health status of a data subject, which reveal information relating to the past, current, or future physical or mental health status of the data subject.^1^

At the European level, the European Commission plans to create a European Health Data Space (EHDS) by 2025. The EHDS aims to promote better data access and exchange by developing an interoperable infrastructure, including HD generated in interactions with healthcare providers, such as electronic health records (EHRs), genomic data, or data from patient registries, as well as individually collected health and certain types of wellness data from smartphones and wearables.^2^

These HD can be used for two purposes: primary use (PU) and secondary use (SU). PU means the utilization of personal HD for the direct delivery of medical care to patients, such as by sharing personal HD with healthcare providers.^3^ As part of the planned EHDS, important care and treatment information, such as diagnoses, therapeutic measures, treatment reports, and medication plans, will be transparently accessible to the patients through their personal health records (PHRs) across European countries.^4^ This availability has various implications for the delivery of medical care. Overall, it can support patients and healthcare professionals in receiving or providing care, such as when moving between EU member states. Furthermore, it can ensure efficient inter- and intrasectoral patient healthcare; avoid overuse, underuse, and misuse of health services; and support diagnostic findings, improving patient safety and care quality.^5–8^ SU means the utilization of pseudonymized or anonymized HD for purposes other than direct patient care, including activities such as research (both commercial and noncommercial), public health monitoring, and healthcare policy development.^3^ It can help to understand patient health, optimize treatment strategies, and support medical research.^9–11^

However, amidst the promise of this digital revolution lies a critical point: the willingness of patients to share their HD for PU and SU. Understanding the factors influencing their decisions in this regard is paramount to realizing the full potential of digital health initiatives.

A few models and theories in the field of data sharing and technology acceptance offer potential predictors for patients’ willingness to share their HD. The most common are the Unified Theory of Acceptance and Use of Technology (UTAUT) model^12,13^ and its follow-up, the UTAUT2 model.^14^ They combine other theories such as Rogers’ Innovation Diffusion Theory (IDT),^15,16^ the Theory of Reasoned Action (TRA),^17^ the Theory of Planned Behavior (TPB),^18^ the Motivational Model (MM),^19^ and the Technology Acceptance Model (TAM),^20–22^ which has been expanded to TAM2.^23^ While these theories and models mainly focus on facilitators, other notable models address potential barriers like privacy and security concerns, such as the User Resistance Model (URM),^24–27^ the Dual Factor Model of IT Usage, which combines the URM and the UTAUT,^28,29^ the Concerns for Information Privacy (CFIP) instrument,^30–36^ and the Internet Users’ Information Privacy Concerns (IUIPC) construct,^37,38^ which became incorporated into the Internet Privacy Concerns (IPC) model^39^ and restructured to create the Health Information Privacy Concern (HIPC) scale.^40^ However, none of these models and theories has explicitly addressed factors influencing patients’ willingness to share their digital HD for PU and SU. Nonetheless, they can serve as a theoretical basis for mapping the available evidence.

In the literature, various types of reviews have examined the available evidence on patients’ willingness to share their digital HD for PU and SU.^41–48^ For example, the literature review by Esmaeilzadeh and Sambasivan^46^ aimed to identify factors that encourage patients to support health information exchange (HIE). A recent review focused on how to implement practical consent by including the patient's perspective in digital health consent.^47^ Another review focused on consent procedures for reusing routinely recorded HD in scientific research.^48^ While there are several existing reviews, they all focus on specific HD types, healthcare technologies, study designs, or countries.

### Aim of this overview of reviews

Against this background, the aim of our work is to provide an international theory- and evidence-based overview of all systematic reviews (SRs) and, thus, primary studies available on factors influencing patients’ willingness to share their digital HD for PU and SU, independent of study design, HD type, and healthcare technology type, qualitatively assessing the evidence.

### Objective

The research question examined in our overview is: What factors influence patients’ willingness to share their digital HD for PU and SU?

## Methods

We conducted an overview of SRs to provide a meta-level synthesis of the aforementioned research question. The corresponding protocol was published in PROSPERO (reference number CRD42023429302) before starting the overview and was kept current. The methods and results are reported according to the Preferred Reporting Items for Overviews of Reviews (PRIOR) statement (see online Appendix 1).^49^

### Inclusion and exclusion criteria

Our inclusion and exclusion criteria were selected using the Population, Concept/Construct, and Context (PCC) framework proposed by Aromataris (Table 1).^50^

**Table 1.** SR inclusion and exclusion criteria according to the PCC framework.

PCC framework item	Inclusion criteria	Exclusion criteria
Population	Adult patients (aged ≥18 years): Defined as all individuals who have been and/or are in contact with the healthcare system in the past and/or present.	
Concept/construct	Any factors influencing patients’ willingness to share their digital HD for PU and SU. Influencing factors reported by patients, including views, attitudes, opinions, perspectives, thoughts, awareness or acceptance of HD sharing.	Opinions from groups other than patients, such as healthcare providers or experts. Only technical methods for analyzing, sharing, and linking HD, such as artificial intelligence and blockchain. Only containing the design of consent forms. Only containing legal or ethical frameworks.
Context	Sharing of digital HD for PU and SU: Defined as sharing of legally protected HD generated during inpatient and outpatient treatment by healthcare providers and is recorded and stored electronically.	Sharing of digital HD collected only by the patients, such as through wearables. Sharing of digital HD collected only during clinical trials.
Review types	SRs: Defined as reviews applying a comprehensive, reproducible search strategy and providing a critical appraisal of study quality.	Overview of reviews.
Further criteria	SRs that are peer-reviewed and available in a full-text format. SRs published between 1 January 2013 and 31 May 2023. No language restrictions.	

PCC: Population, Concept/Construct, and Context; HD: health data; PU: primary use; SU: secondary use.

### Search strategy

We followed a sensitive search approach to identify as many relevant records as possible.^51^ An electronic database search of the MEDLINE and Embase (both via Ovid) and Scopus (via Elsevier) literature databases, complemented by additional searches of Google Scholar, was conducted in June 2023 to identify potentially eligible SRs. The reference lists of the included SRs (backward citation tracking) and the articles citing them (forward citation tracking) were searched in MEDLINE via Ovid. The search strings for all three databases are reported in Appendix 2 in online. They included free-text terms and synonyms as well as corresponding medical subject heading (MeSH) terms containing the construct of willingness (e.g. motivation), covering the context of healthcare provider-related data (e.g. medical record), and referring to HD sharing (e.g. exchange) for PU and SU (e.g. research). Moreover, the publication type was set to SRs only. All single components were linked using the Boolean operator AND. For the population criteria of adult patients, the human filter was set. The time frame was restricted to SRs published between 1 January 2013 and 31 May 2023.

### Study selection

All SRs identified in the electronic searches were imported into EndNote X9 (Clarivate Analytics, Boston, MA, USA), duplicates were removed, and the unique records were uploaded to Rayyan^52^ for the selection process. First, three project team members (SF, CL, and MS) independently screened the titles and abstracts of a randomly selected a 5% subset of records found in an initial search query. The interrater agreement was assessed using Fleiss’ kappa (κ)^53^ and was initially moderate (κ = 0.49). After establishing a more precise common understanding of the inclusion and exclusion criteria, an additional randomly selected 5% subset of records yielded substantially stronger agreement (κ = 0.76). Based on this, one reviewer (SF) screened titles and abstracts and excluded ineligible records. After the title-abstract screening, two reviewers (SF, CL) independently reviewed the full text of the remaining records based on the set inclusion and exclusion criteria (Table 1). The interrater agreement, assessed using Cohen's kappa (κ),^53^ was 0.82, indicating almost perfect agreement.

### Data extraction and analysis

A standardized data extraction sheet was developed in Microsoft Excel (Seattle, WA, USA). Relevant data were extracted according to the established PCC framework. The Excel spreadsheet contained the following information: (1) citation details; (2) review type defined by the authors; (3) search strategy details; (4) publication date range and data collection range if available; (5) number, types, and country of origin of included studies; (6) method(s) of evidence analysis/synthesis; (7) critical appraisal tool(s) used; (8) objective(s); (9) setting/context; (10) participants details; and (11) reported relevant predictors. The data were extracted by one reviewer (SF) and verified for accuracy and completeness by a second reviewer (CL). Any disagreements between reviewers were resolved through discussion or with the assistance of a third reviewer (MS, SD). The extracted data is presented as a table in Appendix 3 in online.

### Quality assessment

The revised measurement tool to assess systematic reviews (R-AMSTAR2) was used to assess the methodological quality of the included SRs and identify critical deficiencies reducing overall confidence. It consists of 16 items covering various aspects, including study selection, data extraction, and risk of bias (RoB). R-AMSTAR2 represents a standardized tool for assessing the transparency and rigor of SR methods, contributing to the reliability and validity of evidence synthesis from randomized or nonrandomized studies of healthcare interventions.^54^ There was no comparable checklist for the focus of our work, which led us to undertake several modifications. Item 9, which asked whether a satisfactory technique has been used to assess RoB in the primary studies included in the SR, was adapted to the context of our research question. We aimed to identify predicting factors at a qualitative level, which will not be provided by randomized controlled trials. Therefore, this item was adapted to better reflect the specifics of assessing RoB for qualitative research. The domains confounding and selection bias were replaced by an examination of whether an adequate quality assessment tool was used for the respective study design and at the individual study level. The critical and noncritical items were counted, and median and interquartile range of the critical items were calculated per SR.

The critical appraisal was pilot-tested on two SRs and then conducted on all others by one reviewer (SF) and verified for accuracy and completeness by a second reviewer (CL). Any disagreements were resolved by discussion or with the assistance of a third reviewer (MS or SD) until a consensus was reached.

### Data synthesis

The results were synthesized by categorizing the relevant predictors influencing patients’ willingness to share their digital HD into cohesive themes and presenting them in a tabular synthesis. The categorized factors were then clustered into main categories. A deductive-inductive approach was used to derive the factors and main categories for PU and SU.

As a theoretical basis for the deductive identification of corresponding factors and categories, existing theories and models on HD sharing and technology acceptance described in the Introduction section were considered. This deductive basis provided 10 factors: costs of data sharing engagement,^14^ age,^12–14^ sex,^12–14^ privacy and security concerns not specified,^30–39^ health status,^12,13^ motivation/interest,^12,13,19^ altruism,^12,13,17,18^ social responsibility,^12,13,17,18^ expected usefulness not specified,^20–23^ and expected ease of use not specified.^20–23^ This process led to eight main categories: sociodemographic factors,^12–14^ privacy and security concerns,^30–39^ facilitating conditions,^12–14^ user resistance reasons,^12,13,24–29^ social influence,^12–14^ expected usefulness,^20–23^ expected ease of use,^20–23^ and previous experience with the healthcare system.^12–16,23^ In the second step, the inductive thematic coding process revealed additional or adapted influencing factors and main categories. The procedure and assignment were discussed and consented to by the review team.

Finally, the direction of influence was determined based on the included SRs’ results and labeled as facilitator, barrier, or inconsistent (for factors that were unclassifiable). The factors were considered consistent facilitators or barriers if all SRs indicated the same direction. Conversely, the factor was considered inconsistent if at least one SR had a conflicting statement. We included all appropriate findings and weighted them equally, regardless of the study type and study quality, to obtain the maximum amount of valuable evidence. Articles included in more than one of the included SRs were only counted once in this overview to avoid bias by double counting.

## Results

### Search results

**Figure 1.**
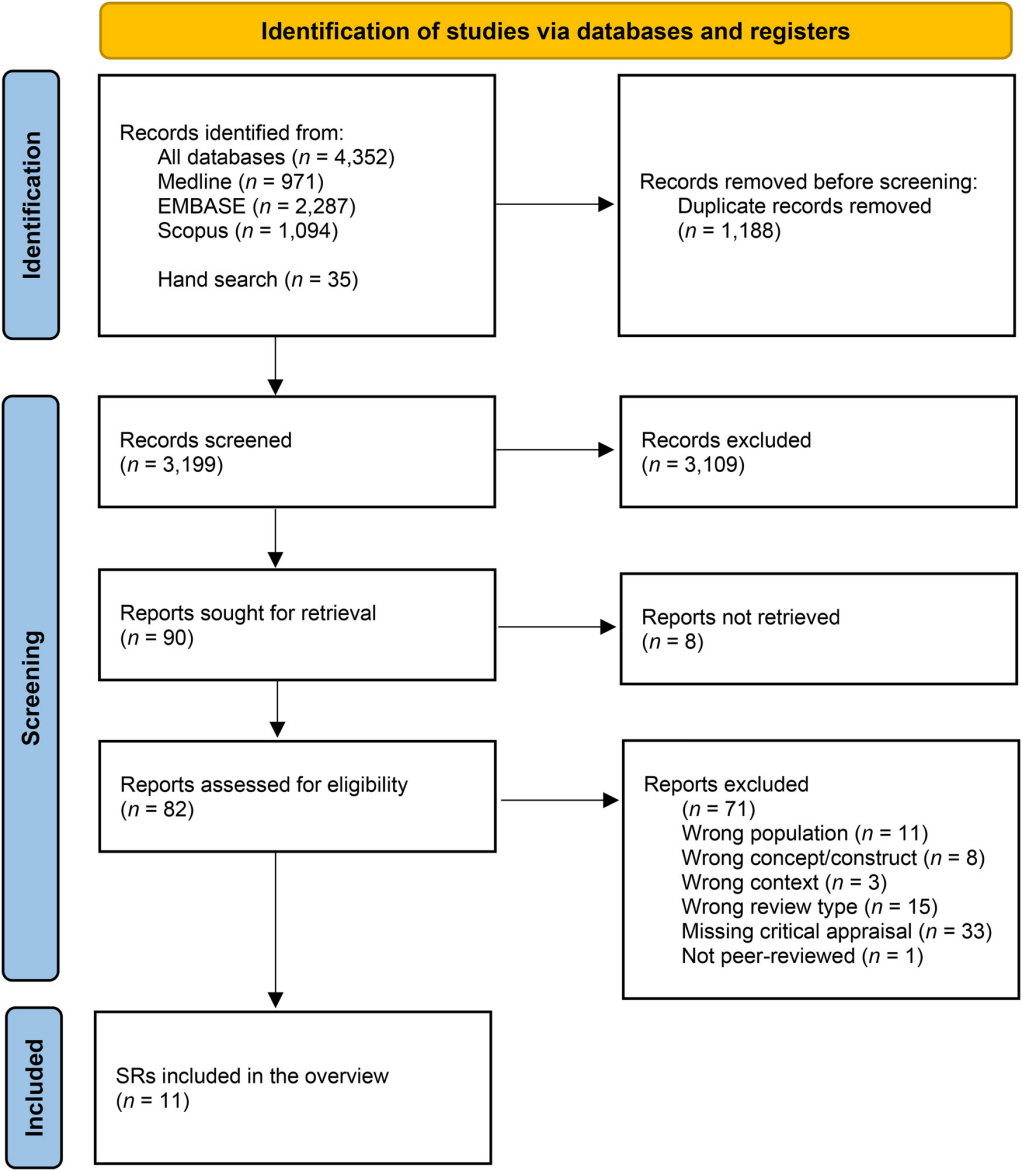
PRISMA flowchart of the SR selection process.

PRISMA: Preferred Reporting Items for Systematic Reviews and Meta-Analyses; SR: systematic review.

The SR selection process is illustrated in Figure 1. The initial search identified 4352 records, with a further 35 potentially relevant records identified by hand searches. After duplicate removal, 3199 of the 4387 records remained, of which the full text was sought for 90. The full text could not be retrieved for eight,^55–62^ leaving 82 eligible for full-text screening. Of these 82 articles, 33 (40%) were missing a critical appraisal of study quality, 15 (18%) did not conduct a systematic literature search, 11 (13%) solely targeted a population that did not meet our inclusion criteria (e.g. healthcare providers or Information Technology staff), and eight (10%) focused on a concept/construct that did not meet our inclusion criteria (e.g. the description of ethical principles or legal frameworks only). The rest was excluded due to wrong context or missing peer-review. A list of the excluded SRs by exclusion criteria is provided in online Appendix 4. Therefore, after excluding these articles, our overview examined 11 SRs.

### Reviews characteristics

The 11 included SRs^63–73^ were published between 2014 and 2021 and examined 321 articles (mean: 25, range: 16–104). Overall, 51/321 (15.9%) references were duplicated once or multiple times across SRs. A list of overlapping articles with the number of duplicates and associated SRs is provided in online Appendix 5.

Regarding the purpose of data sharing, six SRs reviewed articles on PU,^63,65,66,69,70,72^ four SRs reviewed articles on SU,^64,67,68,73^ and one reviewed articles on PU and SU.^71^

Seven SRs^64–70^ did not follow a theory-based approach to evidence synthesis. Overall, the SRs were heterogeneous in participant details (e.g. general public and different types of patients), setting (e.g. inpatient and outpatient settings), and research context (e.g. tethered and untethered EHRs).

### Population

Seven of the 11 SRs included a mix of the general population and patients with different diseases,^65,67–69,71–73^ such as metabolic, rheumatic, renal, cardiac, pulmonary, neurological, psychiatric, musculoskeletal, rare, or genetic diseases. Two SRs were restricted to patients with different types of diabetes (with comorbidities).^65,70^ Five SRs provided information on the total sample size of the primary studies reviewed,^63,67,68,72,73^ which ranged from 97^63^ to 216,149 participants.^68^

### Setting

This overview encompassed different types of healthcare technologies and healthcare settings. Hutchings et al. included general health administrative data.^67,68^ Amante et al.^65^ and Dendere et al.^66^ focused only on patient portals (PPs). O’Connor et al. considered both PHRs and PPs.^72^ All other SRs focused on different types of medical records.^63,64,69–71,73^ The SR by Moon et al. was the most comprehensive regarding the platforms and settings covered. It looked at electronic personal health information, PHRs, and integrated EHRs in clinical primary care, hospital-affiliated clinics, hospital outpatient clinics, general practices, and ambulatory care sites.^71^ Other SRs were limited to clinical settings,^65^ hospital inpatient settings,^66^ or primary care.^69,70^

The characteristics of the included SRs are summarized in Table 2. None performed a meta-analysis due to widespread heterogeneity in study designs, outcomes, population groups, and various reported measures.

**Table 2.** The purpose, objective(s), population(s), setting/context, data collection, and quality assessment of the included SRs.

Reference	Purpose	Objective(s)	Population(s)	Setting/context	Data collection	Quality assessment
Abd-alrazaq et al. (2019) ^63^	PU	Influencing factors for intention to use PHRs Analyzing the initial use rate of PHRs	General public and patients with different conditions (e.g. diabetes, HIV, and rheumatic diseases)	Tethered PHRs, connected with EMRs	No. of databases: 42 No. of papers: 104 (97 studies) Published: 2006–2018	Mixed methods appraisal tool (MMAT)
Aitken et al. (2016) ^64^	SU	Evaluating key issues of patients’ responses in data sharing and data linkage for research	Patients, lay persons and the general public	PCHRs, other existing medical data or health material	No. of databases: 5 No. of papers: 25 (36 studies) Published: 2002–2014	Critical Appraisal Skills Programme (CASP)
Amante et al. (2014) ^65^	PU	Characteristics of PP users Barriers and facilitators of PP enrollment and utilization	Patients with diabetes	Clinically integrated PPs	No. of databases: 2 No. of papers/studies: 16 Published: 2007–2016	Mixed Methods Appraisal Tool (MMAT)
Dendere et al. (2019) ^66^	PU	Identification of influencing factors and best practices for successful implementation of EMRs	Patients with different conditions (e.g. cancer, HIV, and cardiac diseases)	Electronic PPs tethered to EMRs in hospital inpatient setting	No. of databases: 3 No. of papers: 58 (555 studies) Published: 2005–2018	Revised measurement tool to assess systematic reviews (AMSTAR2) and Quality Assessment Tool for Studies with Diverse Designs (QATSDD)
Hutchings et al. (2020) ^67^	SU	Patients’ concerns about privacy, trust, and transparency for health administrative and clinical trial data reuse	General public and patients with different conditions (e.g. mental health illnesses and hypertension)	Health administrative data	No. of databases: 8 No. of papers/studies: 35 Published: 1997–2020	QualSyst-Criteria (acc. to Kmet et al.)
Hutchings et al. (2021) ^68^	SU	Revealing attitudes on consent and the use of health administrative data for research	General public and patients with different conditions (e.g. mental health illnesses and hypertension)	Health administrative data	No. of databases: 8 No. of papers/studies: 47 Published: 1998–2020	QualSyst-Criteria (acc. to Kmet et al.)
Mold et al. (2015) ^69^	PU	Information on patients’ attributes and variations concerning access and use of EHRs	Users and nonusers of online record access and remote contact services, including patients	Online health record access and transactional services in primary care	No. of databases: 10 No. of papers/studies: 17 Published: 2003–2012	Risk of bias (RoB)
Mold et al. (2018) ^70^	PU	Characteristics of CMR users Challenges, barriers, and impacting system features on CMR use	Users and nonusers of CMRs, including adults with type 2 diabetes (combined with hypertension) and their caregivers	CMRs in primary care	No. of databases: 9 No. of papers: 28 (37 studies) Published: 2004–2015	Mixed Methods Appraisal Tool (MMAT)
Moon et al. (2017) ^71^	PU and SU	Information about patient’s data sharing preferences Influencing factors associated with HIE	Patients and the general public	HIE of ePHIs, PHRs, and integrated EHRs in clinics primary care, hospital-affiliated clinics, hospital outpatient clinics, general practices, and ambulatory care sites	No. of databases: 2 No. of papers: 18 (16 studies) Published: 2009–2015	Olsen and St George's (2004) Cross-sectional study design and data analysis framework and Kuper, Lingard, and Levinson (2008) Critically appraising qualitative research strategy
O’Connor et al. (2016) ^72^	PU	Assessing factors, including barriers and facilitators, affecting engagement and enrollment in DHI	Patients and healthy individuals	PHRs or PPs in primary, secondary or tertiary care, the home or workplace	No. of databases: 6 No. of papers/studies: 19 Published: 2005–2015	Enhancing transparency in reporting the synthesis of qualitative research (ENTREQ)
Stockdale et al. (2019) ^73^	SU	Patients’ views on data sharing for research Patients’ preferences on the consent model	General public and patients with different conditions (e.g. cancer and multiple sclerosis)	Data from electronic hospital records and electronic general practice records	No. of databases: 3 No. of papers/studies: 20 Published: 2006–2016	Mixed Methods Appraisal Tool (MMAT)

PU: primary use; PHR: personal health record; EMR: electronic medical record; SU: secondary use; PCHR: personally controlled health record; PP: patient portal; EHR: electronic health record; CMR: computerized medical record; HIE: health information exchange; ePHI: electronic personal health information; DHI: digital health intervention.

### Characteristics of the included papers

#### Countries

The 321 individual articles reviewed in the 11 included SRs originated from four continents, where the research was conducted (Table 3). Most originated in the United States and Canada (216/321, 67.3%),^63–72^ followed by Europe (83/321, 25.9%),^63,64,66–73^ Australia and New Zealand (11/321, 3.4%),^63,67,68,70^ and Asia (9/321, 2.8%).^63,64,66–68,71^ One article in the SR by Stockdale et al.^73^ stated no country, and one in SR by Aitken et al.^64^ indicated countries worldwide.

**Table 3.** Number of papers by continent and country with the related SRs.

Continent and country	No. of papers	% of papers	Related SRs
**The United States of America and Canada**	216	67.3	
The United States of America	192	59.8	^63–72^
Canada	22	6.9%	^63,64,66–68,72^
Argentina	2	0.6	^63,66^
**Europe**	83	25.9	
United Kingdom	54	16.8	^64,66–73^
Netherlands	7	2.2	^63,66,70^
Norway	6	1.9	^66,69,72^
Europe not specified	4	1.2	^67,68^
Sweden	3	0.9	^64,66^
Finland	2	0.6	^63,67,68^
Germany	2	0.6	^66,68^
Belgium	1	0.3	^67,68^
Portugal	1	0.3	^63^
Spain	1	0.3	^72^
Austria	1	0.3	^66^
France	1	0.3	^70^
**Australia and New Zealand**	11	3.4	
Australia	7	2.2	^67,68,70^
New Zealand	4	1.2	^63,67,68^
**Asia**	9	2.8	
Korea	3	0.9	^63,66,71^
China	2	0.6	^67,68^
Japan	2	0.6	^64,67^
Taiwan	1	0.3	^68^
Jordan	1	0.3	^63^
**Worldwide**	1	0.3	^64^
Not stated	1	0.3	^73^

SR: systematic review.

#### Study designs

The research methodology of the studies reported in the 321 articles varied between quantitative (observational and interventional studies) and qualitative study designs. Most used a quantitative design (158/321, 49.2%), followed by a qualitative design (108/321, 33.6%), mixed-method design (40/321, 12.5%), and review design (16/321, 5.0%). Table 4 summarizes the characteristics in terms of included research designs and provides information on data analysis and (theory-based) synthesis approaches of the 11 included SRs.

**Table 4.** The research designs of included papers and the data analysis and synthesis approaches of the 11 included SRs.

Reference	Research designs	Data analysis and synthesis approach	Theory-based synthesis
Abd-alrazaq et al. (2019)^63^	Method: Quantitative (90) Qualitative (10) Mixed-methods (4) Design: Cross-sectional Cohort Case-control	Narratively; categorization of factors with framework method	Yes
Aitken et al. (2016)^64^	Method: Qualitative (16) Mixed-methods (6) Literature/policy/systematic review (3) Design: Focus groups Interviews Deliberative events Dialogue workshops Surveys	Narratively; deductive-inductive thematic synthesis	No
Amante et al. (2014)^65^	Method: Quantitative (9) Mixed-methods (4) Qualitative (3) Design: RCT Cross-sectional Cohort Focus groups	Quantitative results: mathematical statistics Qualitative results: categorization of factors	No
Dendere et al. (2019)^66^	Method: Qualitative (19) Quantitative (18) Reviews (12) Mixed-methods (9) Design: Surveys Focus groups Interviews Quasi-experimental	Narratively; categorization of themes	No
Hutchings et al. (2020)^67^	Method: Qualitative (28) Mixed-methods (7) Design: Face-to-face interviews and/or focus groups Surveys Deliberative events + surveys/ + focus groups + interviews Citizens jury model Cohort + RCT Other	Quantitative results: descriptive statistics Qualitative results: meta-aggregative approach with framework method	No
Hutchings et al. (2021)^68^	Method: Quantitative (31) Qualitative (8) Mixed-methods (8) Design: Face-to-face/telephone/questionnaire-based interviews Focus groups (+ interviews) Surveys (+ focus groups) Deliberative events + surveys Citizens jury model (Cohort +) RCT Other	Quantitative results: descriptive statistics Qualitative results: meta-aggregative approach with framework method	No
Mold et al. (2015)^69^	Method: Quantitative (16) Mixed-methods (1) Design: RCT (with qualitative element) Cohort Cluster RCT Quasi experimental trial, nonrandomized	Quantitative results: mathematical statistics Qualitative results: narratively	No
Mold et al. (2018)^70^	Method: Quantitative (15) Qualitative (11) Mixed-methods (1) Interpretative review (1) Design: Audits Retrospective, prospective and longitudinal cohort Quasi-experimental design Focus groups/interviews RCT Surveys	Quantitative results: descriptive and mathematical statistics Qualitative results: categorization of themes with framework method	No
Moon et al. (2017)^71^	Method: Quantitative (13) Qualitative (5) Design: Surveys Interviews	Narratively; clustering of factors	Yes, authors created their own framework
O’ Connor et al. (2016)^72^	Method: Qualitative (15) Mixed-methods (4) Design: Mixed interventions Interviews Focus groups Participant observations Documentary evidence Surveys	Content analyzing methods	Yes, authors created their own framework
Stockdale et al. (2019)^73^	Method: Qualitative (10) Quantitative (7) Mixed-methods (3) Design: Focus groups Interviews Surveys Workshops Other	Quantitative results: descriptive statistics Qualitative results: narratively; content analyzing methods	Yes

RCT: randomized controlled trial.

### Factors influencing patients’ willingness to share their digital HD for PU and SU

#### Overview

The deductive-inductive thematic coding process revealed 41 factors grouped into 15 main categories influencing patients’ willingness to share their HD, indicating the direction of influence. Among them, 16 (39%) factors were related to PU only, six (15%) were related to SU only, and 19 (46%) factors were related to both PU and SU.

Ten (24%) of the 41 influencing factors and eight (53%) of the 15 main categories were deductively derived, as described in the Methods section. The remaining 31 (76%) of the 41 influencing factors and seven (47%) of the 15 main categories were inductively derived based on the evidence found.

#### PU

Thirty-five factors influencing patients’ willingness to share their digital HD for PU were identified: 22 as facilitators (e.g. higher income), nine as barriers (e.g. high costs for participation), and four as inconsistent (e.g. sex). Table 5 presents the detailed results on the facilitators, barriers, and inconsistent factors with underlying models/theories.

**Table 5.** The main categories and influencing factors on HD sharing for PU with the corresponding direction of influence and category development with underlying models/theories.

Main category (number of appearances in the included SRs)	Influencing factor (number of appearances in the included SRs)	Direction of influence: facilitator/barrier/inconsistent with corresponding SRs	Sharing type	Category development with underlying models/ theories
Socioeconomic factors				Inductively
	Education (*n* *=* 7)	(+) Higher educational level ^63,65,67,68,70,71,73^	PU & SU	Inductively
	Income (*n* = 5)	(+) Higher income ^63,65,66,70,71^	PU	Inductively
	Socioeconomic factors not specified (*n* = 4)	(+) Higher socioeconomic status ^63,69^ with regard to PU	PU & SU	Inductively
	Health insurance status (*n* = 4)	(+) Existing/commercial health insurance status ^65,68–70^	PU & SU	Inductively
	Residential area (*n* = 3)	(+) Living in wealthier neighborhoods ^63,68,70^	PU & SU	Inductively
	Employment status (*n* = 3)	(+) (Full-time) employed ^63,70,71^	PU	Inductively
	Patient costs of data sharing engagement (*n* = 1)	(−) High costs for participation ^72^	PU	Deductively from UTAUT2 ^14^
Sociodemographic factors				Deductively from UTAUT2 ^14^/UTAUT ^12,13^
	Age (*n* = 8)	(+/−) Inconsistent direction ^65–71,73^	PU & SU	Deductively from UTAUT2 ^14^/ UTAUT ^12,13^
	Sex (*n* = 8)	(+/−) Inconsistent direction ^63,65–71^	PU & SU	Deductively from UTAUT2 ^14^/ UTAUT ^12,13^
	Ethnicity (*n* = 8)	(+/−) Inconsistent direction ^63,65,66,68–71,73^	PU & SU	Inductively
	Family situation (*n* = 2)	(+) Married, children in the household, caregivers presence ^63,71^	PU	Inductively
Privacy and security concerns				Deductively from CFIP^30–36^/IUIPC ^37,38^/IPC ^39^
	Privacy and security concerns not specified (*n* = 8)	(−) Existing general privacy and security concerns ^63–67,71–73^	PU & SU	Deductively from CFIP ^30–36^/ IUIPC ^37,38^/ IPC ^39^
	Concerns about data processing (*n* = 5)	(−) Existing privacy and security concerns about data processing ^64,67,68,71,73^	PU & SU	Inductively
	Concerns about data access (*n* = 2)	(−) Existing privacy and security concerns about data access ^67,73^	PU & SU	Inductively
Knowledge and understanding				Inductively
	Technical/digital literacy (*n* = 4)	(+) Greater technical/digital literacy ^65,67,68,72^	PU & SU	Inductively
	Health literacy (*n* = 4)	(+) Greater health literacy ^65,68,70,72^	PU & SU	Inductively
	Awareness of PHR (-features) (*n* = 3)	(−) Lack of awareness of PHR (-features) ^63,65,70^	PU	Inductively
Health conditions				Inductively
	Health status (*n* = 8)	(+/−) Inconsistent direction ^63,65–71^	PU & SU	Deductively from adapted UTAUT ^12,13^
	Utilization of healthcare services (*n* = 2)	(+) Regular/higher utilization of healthcare services ^65,71^	PU	Inductively
Expected usefulness				Deductively from TAM2 ^23^/TAM ^20–22^
	Expected usefulness not specified (*n* = 3)	(+) Expected usefulness in general ^63,64,66^	PU & SU	Deductively from TAM2 ^23^/TAM ^20–22^
	Information, organization, and documentation (*n* = 2)	(+) Improved own information, organization and documentation ^66,71^	PU	Inductively
	Communication (*n* = 2)	(+) Improved communication ^66,71^	PU	Inductively
	Medical care (*n* = 1)	(+) Better medical care ^71^	PU	Inductively
Facilitating conditions				Deductively from UTAUT2 ^14^/UTAUT ^12,13^
	Trust, confidentiality and transparency (*n* = 5)	(+) Assurance of trust, confidentiality, and transparency ^64,67,68,71,73^	PU & SU	Inductively
	Autonomy and control (*n* = 3)	(+) Assurance of autonomy and control ^64,68,71^	PU & SU	Inductively
Social influence				Deductively from UTAUT2 ^14^/UTAUT ^12,13^
	Family and peers (*n* = 3)	(+) Recommendation/support from family and peers ^65,68,72^	PU & SU	Inductively
	Healthcare professionals (*n* = 3)	(+) Recommendation/encouraging behavior by healthcare professionals ^66,67,72^	PU	Inductively
Previous experience with healthcare system				Deductively from UTAUT2 ^14^/UTAUT ^12,13^/TAM2 ^23^/IDT ^15,16^
	Technical aspects (*n* = 3)	(−) Negative digital/data sharing experience ^65,70,72^	PU	Inductively
	Personal aspects (*n* = 2)	(+) Current dissatisfaction and negative experience with traditional healthcare ^65,72^	PU	Inductively
Technological conditions				Inductively
	Computer/internet access (*n* = 3)	(+) Available computer/internet access ^63,65,72^	PU	Inductively
	Internet use (*n* = 2)	(+) Internet use ^63,71^	PU & SU	Inductively
Expected ease of use				Deductively from TAM2 ^23^/TAM ^20–22^
	Expected ease of use not specified (*n* = 4)	(+) Expected usability of EHR ^63,65,66,72^	PU	Deductively from TAM2 ^23^/TAM ^20–22^
User resistance reasons				Deductively from dual factor model of IT usage ^28,29^/ URM ^24–27^/UTAUT ^12,13^
	Concerns about being discriminated or stigmatized (*n* = 4)	(−) Existing concerns about being discriminated or stigmatized ^64,68,71,73^	PU & SU	Inductively
Lifestyle aspects				Inductively
	Lifestyle aspects not specified (*n* = 2)	(−) Busy lifestyle ^65,72^	PU	Inductively
Personality traits				Inductively
	Motivation/ interest (*n* = 2)	(−) Lack of motivation to understand or improve health/no interest in changes ^65,72^	PU	Deductively from UTAUT ^12,13^/ MM ^19^

SR: systematic review; PU: primary use; SU: secondary use; UTAUT/UTAUT2: Unified Theory of Acceptance and Use of Technology; CFIP: Concern for Information Privacy; IUIPC: Internet Users’ Information Privacy Concerns; IPC: Internet Privacy Concerns; MM: Motivational Model; PHR: personal health record; URM: User Resistance Model; TAM/TAM2: Technology Acceptance Model; EHR: electronic health record; IDT: Innovation Diffusion Theory.

#### SU

Twenty-five factors influencing patients’ willingness to share their digital HD for SU were identified: 13 as facilitators (e.g. assurance of autonomy and control), six as barriers (e.g. existing concerns about commercial use), and six as inconsistent (e.g. ethnicity). Table 6 presents the detailed results on the facilitators, barriers, and inconsistent factors with underlying models/theories.

**Table 6.** The main categories and influencing factors on HD sharing for SU with the corresponding direction of influence and category development with underlying models/theories.

Main category (number of appearances in the included SRs)	Influencing factor (number of appearances in the included SRs)	Direction of influence: facilitator/barrier/inconsistent with corresponding SRs	Sharing type	Category development with underlying models/ theories
Sociodemographic factors				Deductively from UTAUT2 ^14^/UTAUT ^12,13^
	Age (*n* = 8)	(+/−) Inconsistent direction ^65–71,73^	PU & SU	Deductively from UTAUT2 ^14^/UTAUT ^12,13^
	Sex (*n* = 8)	(+/−) Inconsistent direction ^63,65–71^	PU & SU	Deductively from UTAUT2 ^14^/UTAUT ^12,13^
	Ethnicity (*n* = 8)	(+/−) Inconsistent direction ^63,65,66,68–71,73^	PU & SU	Inductively
Socioeconomic factors				Inductively
	Education (*n* = 7)	(+) Higher educational level ^63,65,67,68,70,71,73^	PU & SU	Inductively
	Socioeconomic factors not specified (*n* = 4)	(+/−) Inconsistent direction ^68,73^ with regard to SU	PU & SU	Inductively
	Health insurance status (*n* = 4)	(+) Existing/commercial health insurance status ^65,68–70^	PU & SU	Inductively
	Residential area (*n* = 3)	(+) Living in wealthier neighborhoods ^63,68,70^	PU & SU	Inductively
Privacy and security concerns				Deductively from CFIP ^30–36^/IUIPC ^37,38^/IPC ^39^
	Privacy and security concerns not specified (*n* = 8)	(−) Existing general privacy and security concerns ^63–67,71–73^	PU & SU	Deductively from CFIP ^30–36^ /IUIPC ^37,38^/IPC ^39^
	Concerns about data processing (*n* = 5)	(−) Existing privacy and security concerns about data processing ^64,67,68,71,73^	PU & SU	Inductively
	Concerns about data access (*n* = 2)	(−) Existing privacy and security concerns about data access ^67,73^	PU & SU	Inductively
Facilitating conditions				Deductively from UTAUT2 ^14^/UTAUT ^12,13^
	Trust, confidentiality and transparency (*n* = 5)	(+) Assurance of trust, confidentiality and transparency ^64,67,68,71,73^	PU & SU	Inductively
	Autonomy and control (*n* = 3)	(+) Assurance of autonomy and control ^64,68,71^	PU & SU	Inductively
	Consent management (*n* = 3)	(+/−) Inconsistent direction ^64,68,73^	SU	Inductively
User resistance reasons				Deductively from dual factor model of IT usage ^28,29^/URM ^24–27^/UTAUT ^12,13^
	Concerns about being discriminated or stigmatized (*n* = 4)	(−) Existing concerns about being discriminated or stigmatized ^64,68,71,73^	PU & SU	Inductively
	Concerns about commercial use (*n* = 4)	(−) Existing concerns about commercial use ^64,67,68,73^	SU	Inductively
	Concerns about being overstrained (*n* = 1)	(−) Existing concerns about being overstrained ^68^	SU	Inductively
Health conditions				Inductively
	Health status (*n* = 8)	(+/−) Inconsistent direction ^63,65–71^	PU & SU	Deductively from adapted UTAUT ^12,13^
Knowledge and understanding				Inductively
	Technical/digital literacy (*n* = 4)	(+) Greater technical/digital literacy ^65,67,68,72^	PU & SU	Inductively
	Health literacy (*n* = 4)	(+) Greater health literacy ^65,68,70,72^	PU & SU	Inductively
Personality traits				Inductively
	Social responsibility (*n* = 4)	(+) High social responsibility ^64,67,68,73^	SU	Deductively from UTAUT ^12,13^/ TPB^g^ ^18^/TRA^h^ ^17^
	Altruism (*n* = 2)	(+) Positive altruistic attitude ^67,73^	SU	Deductively from UTAUT ^12,13^ /TPB ^18^/TRA ^17^
Expected public benefit				Inductively
	Expected public benefit not specified (*n* = 3)	(+) Expected public benefit on healthcare ^64,68,71^	SU	Inductively
Expected usefulness				Deductively from TAM2 ^23^/TAM ^20–22^
	Expected usefulness not specified (*n* = 3)	(+) General expected usefulness ^63,64,66^	PU & SU	Deductively from TAM2 ^23^/TAM ^20–22^
Social influence				Deductively from UTAUT2 ^14^/UTAUT ^12,13^
	Family and peers (*n* = 3)	(+) Recommendation/support from family and peers ^65,68,72^	PU & SU	Inductively
Technological conditions				Inductively
	Internet use (*n* = 2)	(+) Internet use ^63,71^	PU & SU	Inductively

SR: systematic review; PU: primary use; SU: secondary use; UTAUT/UTAUT2: Unified Theory of Acceptance and Use of Technology; CFIP: Concern for Information Privacy; IUIPC: Internet Users’ Information Privacy Concerns; IPC: Internet Privacy Concerns; TPB: Theory of Planned Behavior; TRA: Theory of Reasoned Action; URM: User Resistance Model; TAM/TAM2: Technology Acceptance Model.

Online Appendix 6 contains more detailed information on the main categories and individual influencing factors for HD sharing for PU and SU.

### Quality assessment

In summary, the overall confidence rating showed that all 11 included SRs were of critically low quality.^54^ Here, a brief narrative summary is provided on the number of critical items, where the overall median was 3 (interquartile range: 2–4). Six SRs^64–66,70–72^ did not explicitly state that the review methods were established before the review with a written protocol, such as publication in PROSPERO (Item 2). Regarding whether the review authors used a comprehensive literature search strategy (Item 4), only one SR^63^ scored yes, while the other SRs scored partially yes. None of the SRs provided a complete list of potentially relevant studies excluded during the full-text eligibility screening (Item 7). Three SRs that included nonrandomized intervention studies^63,64,72^ did not use a satisfactory technique to assess RoB at the individual study level (adjusted Item 9b). As no meta-analysis was performed within the included SRs, Item 11 on appropriate methods for statistical combination of the results and Item 15 on investigated publication bias were not applicable. Only one SR^63^ considered the RoB of individual studies when interpreting and discussing their results (Item 13). The detailed quality assessments using the R-AMSTAR2 checklist are presented in Table 7.

**Table 7.** Final R-AMSTAR2 checklist results for the 11 included SRs.

Critical items		*		*			*		*			*			*		*		Critical	Noncritical	Rating overall confidence
Item	1	2	3	4	5	6	7	8	9a	9b	10	11a	11b	12	13	14	15	16			
Reference	PICO used?	**Protocol? Significant deviations justified?**	Study design selection explained?	**Comprehensive literature search strategy?**	Study selection duplicate?	Data extraction duplicate?	**List of excluded studies provided? Exclusions justified?**	Studies described in adequate detail?	**Risk of bias assessed appropriately?**		Sources of funding of included studies?	**Appropriate methods?**		RoB in meta-analyses?	**RoB in interpretation?**	Heterogeneity explained?	**Publication bias investigated?**	Conflict of interest?			
Abd-alrazaq et al. (2019) ^63^	y	p	n	y	y	y	n	n	o	n	n	m	m	m	y	n	m	y	2	4	critically low
Aitken et al. (2016) ^64^	y	n	n	p	y	y	n	n	o	n	n	m	m	m	n	n	m	y	4	4	critically low
Amante et al. (2014) ^65^	y	n	n	p	n	n	n	p	y	y	n	m	m	m	n	n	m	y	3	5	critically low
Dendere et al. (2019) ^66^	y	n	n	p	y	n	n	n	o	y	n	m	m	m	n	n	m	y	3	5	critically low
Hutchings et al. (2020) ^67^	y	p	n	p	n	y	n	p	o	y	n	m	m	m	n	n	m	y	2	4	critically low
Hutchings et al. (2021) ^68^	y	p	n	p	y	y	n	p	y	y	n	m	m	m	n	n	m	y	2	3	critically low
Mold et al. (2015) ^69^	y	y	n	p	y	y	n	n	y	y	n	m	m	m	n	n	m	y	2	4	critically low
Mold et al. (2018) ^70^	y	n	n	p	y	y	n	p	y	y	n	m	m	m	n	n	m	y	3	3	critically low
Moon et al. (2017) ^71^	y	n	n	p	n	n	n	p	o	y	n	m	m	m	n	n	m	y	3	5	critically low
O’Connor et al. (2016) ^72^	y	p	n	p	y	y	n	y	o	n	n	m	m	m	n	n	m	y	3	3	critically low
Stockdale et al. (2019) ^73^	y	n	n	p	y	n	n	p	o	y	n	m	m	m	n	n	m	y	3	4	critically low
Total	1	2	3	4	5	6	7	8	9a	9b	10	11a	11b	12	13	14	15	16			
Yes	11	1	0	1	8	7	0	1	4	8	0	0	0	0	1	0	0	11			
Partial Yes	-	4	-	10	-	-		6	0	-	-	-	-	-	-	-	-	-			
No	0	6	11		3	4	11	4	0	3	11	0	0	0	10	11	0	0			
N/A	-	-	-	-	-	-	-	-	7	-	-	11	11	11	-	-	11	-			

The bottom rows list the total number of SRs with R-AMSTAR2 items rated as yes, partial yes, no, and not applicable; hyphens indicate that the answer was not an option for the particular question. Critical domains are marked with an asterisk (*). The last three columns count the number of critical and noncritical flaws in the SR and show an overall confidence rating. Item 9b was adapted to our research question by assessing whether a properly developed rating instrument was used (depending on the included study design). y: yes; n: no; p: partially yes; m: no meta-analysis; o: only nonrandomized intervention studies; N/A: not applicable.

## Discussion

### Principal findings

Our overview aimed to summarize and qualitatively assess the current evidence on factors influencing patients’ willingness to share their HD for PU and SU. Overall, 41 factors were identified and grouped into 15 main categories: 35 factors were identified as influencing HD sharing for PU, and 25 were identified as influencing HD sharing for SU.

A comparison of the respective factors showed that those influencing PU are more individualized, focusing on direct patient care and personal motivations. In contrast, those influencing SU emphasize broader societal benefits, altruism, and diverse user resistance factors. Specifically, it was shown that assurance of trust, confidentiality, transparency, autonomy, and control over data are facilitators for PU and SU. In contrast, existing concerns about data processing and access were barriers for PU and SU. However, consent management was found to be important for SU, reflecting an additional layer of data governance concerns.

Regarding user resistance, concerns about discrimination or stigmatization were barriers for PU and SU. Further barriers to SU were concerns about commercial use and being overstrained, indicating a broader spectrum of resistance factors.

In terms of previous experiences with the healthcare system, technical aspects of digital data sharing and personal aspects of traditional healthcare were relevant for PU, while no such factors were identified as relevant for SU.

Diverse socioeconomic factors were identified as influencing HD sharing for PU. While education, unspecified socioeconomic factors, health insurance status, and residential area were associated with PU and SU, income, employment status, and patient costs of datasharing engagement were only relevant to PU.

Altogether, our findings are crucial to designing and developing digital health policy and initiatives. For this purpose, those factors that can be influenced and that we have identified as consistent predictors have the greatest implications and potential impact. These factors include, among others, individual usefulness and ease of use, the two central variables of the TAM, and privacy and security concerns, which are addressed in several theories, such as the URM or CFIP.

Our overview found that expected usefulness and public benefit were consistent facilitators of patients’ willingness to share their HD for PU and SU, underscoring that perceived value is considered the strongest predictor of EHR adoption and the associated potential of HD sharing.^74^ Therefore, we believe it is crucial to clearly communicate the benefits of PU and SU to patients, especially to regular or frequent users of healthcare services, who would benefit the most. Naturally, this is often related to chronic conditions and older adults. However, health status and age did not exhibit consistent in our overview, while other studies confirmed that lower age and having chronic conditions were positively associated with PP access.^75,76^ Notably, age classification was mainly not based on numbers.^65–71,73^ In particular, recommendations and encouragement from healthcare professionals,^65,66,72^ the promise of increased convenience,^71^ and “open, honest digital interaction” ^72^ can positively impact PHR adoption. Healthcare providers play a key role as change agents in patients’ willingness to use EHRs,^22^ and other studies have suggested that physicians’ encouragement increases their use.^77,78^ Our overview supports this suggestion, with recommendations and encouragement from healthcare professionals identified as consistent facilitators. Consequently, they should be empowered in their role as multipliers to inform patients about the benefits and risks of HD sharing for PU and SU. In addition, extensive public relations work is needed to explain the high added value and low risks of HD sharing, ensured by measures making HD sharing as safe as technically possible.

The lack of awareness of EHR (features) among patients and present concerns about being overstrained were identified as consistent barriers to EHR adoption and HD sharing in our overview and elsewhere.^79–82^ Another review confirmed low participant knowledge of the specific use of routinely collected patient data and low understanding of medical research^73^ as causes of anxiety or feelings of being overwhelmed.^68^ However, increasing understanding can significantly support participation in HD sharing for PU and SU.^83^ Therefore, we recommend disseminating target group-orientated and easily understandable information, such as through trustworthy patient organizations, health insurance companies, and public campaigns. The planned purpose, data protection guidelines, and the option to give or withdraw consent regarding HD sharing should be made available transparently and in simple language. Providing easy-to-understand information would help patients with lower socioeconomic status, health literacy, or technical/digital literacy to be better informed and make informed decisions.

Our overview demonstrates that patients with lower socioeconomic status or health literacy require special attention. These groups should be specifically targeted, such as by providing modular support,^84^ to understand the available digital health information.^85,86^ In addition, providing patients with limited technical knowledge with customized training before using a PP can significantly improve participation in HD sharing for PU.^87^ Moreover, technical assistance ranked first among promoters of HIE adoption.^88^ Furthermore, a lack of understanding between the terms of anonymization and identifiable data negatively influenced HD sharing for SU.^67^ Therefore, public education on national privacy laws and regulations would be beneficial.^67,68^

Our overview also identified expected ease of use as a consistent facilitator of HD sharing for PU. Usability ranked third among promoters of HIE adoption,^88^ and usability elements might be crucial to increase adherence to PP.^89^ User friendliness can be achieved by involving end users from the beginning through a human-centered design process.^90^ While age showed heterogeneous results in our overview, one possible reason for a lower willingness to share HD among older adults could be that EHRs are not sufficiently designed to meet their needs.^91,92^

The last essential aspects to be discussed are privacy and security concerns. They were frequently studied and identified as consistent barriers to HD sharing for PU and SU. However, more negative statements were raised regarding SU. In addition, lower socioeconomic status correlated with greater concerns about privacy.^73^ In this context, assurances about autonomy and control, as well as trust, confidentiality, and transparency, act as consistent facilitators. While broad public support for HD sharing for research has been reported, this was linked to three combined core conditions: protected privacy and security, established trust, and ensured transparency.^45^ Perceived healthcare quality was associated with lower privacy concerns, which can be mitigated by the possible benefits of HIE and positively framed arguments.^33,93^ It has been assumed that societal benefits might outweigh privacy concerns,^67^ and benefits to the population, such as increased understanding and better treatment of diseases, may outweigh potential risks and concerns, positively influencing HIE.^71^

### Discussion on reviews characteristics

Our overview included various types of healthcare technologies, such as EHRs and PPs, in both inpatient and outpatient settings, as the healthcare technology landscape is not “one-size-fits-all.” Different functionalities and features that capture individual needs and preferences will most likely influence the decision to use PHRs.^94^ The most common type worldwide, tethered PHRs (also called PPs), gives patients no or only partial control over their medical records within a particular healthcare network.^95,96^ In contrast, untethered PHRs enable patient access and control, providing patients with comprehensive health management.^63^ Untethered PHRs allow users to manually enter and manage their health information, providing greater autonomy. Assurance of autonomy and individual control over personal data have been found to facilitate HD sharing.^64^

Most of the included SRs focused only on factors influencing the intention to use EHRs, while others also included the initial use^63,65,72^; however, none focused on long-term use. According to Abd-alrazaq et al.,^63^ factors influencing intention to use do not necessarily influence actual use and vice versa. As the success of health technologies and the subsequent effectiveness of various outcomes depend on ongoing utilization,^97,98^ it would be interesting to conduct longitudinal studies and SRs on active use over time.

The type of HD to be shared for PU and SU varied across individual SRs and, in some cases, was not specified in detail or considered in a differentiated manner. In addition, the distinction between HD collected for administrative purposes and electronic documentation in EHRs was less clear.^67^ Patients’ willingness to share their HD may also be strongly influenced by the type of HD involved. Willingness to consent to medical record access for research was lower for sensitive topics, such as sexual orientation, sexual or sexually transmitted diseases, infectious diseases, reproductive issues, contraception or female genital disorders, urinary disorders, mental health, disability, and genetics.^68^ Moreover, Moon et al. demonstrated greater willingness to share nonsensitive than sensitive digital HD and mental health, drug/alcohol use, and sexually transmitted diseases were considered particularly sensitive.^71^ This observation may explain the inconsistent findings on preferred consent models and highlights the importance of personal granular control over access, limited data set, restriction of specific information, and choice in HD sharing.^71^

Our overview deliberately aimed to provide an international perspective on the factors influencing HD sharing. Most primary studies (60%) were conducted in the United States. Therefore, some findings may not be generalizable to other countries (e.g. those in Europe) due to different legal regulations in healthcare, health systems, and health funding models. The unified European approach to HD governance is characterized by comprehensive regulatory frameworks that prioritize individual rights and stronger data protection,^99^ addressing patients’ privacy and security concerns. It is based on the GDPR, which sets strict requirements for HD sharing. The GDPR classifies HD as a special category of personal data with enhanced protections.^100^ Transparency regarding SU is guaranteed, which was identified as a modifiable facilitator in the included SRs and elsewhere.^101,102^ At the same time, the EU countries have options for specific implementation (e.g. concerning various consent models). At the European level, complementing the GDPR, the EHDS regulation was entered into force on 26 March 2025 with different key milestones until 2034 toward full implementation.^103^ Key principles addressed include granular control over personal HD, secure data exchange, interoperability through common standards, and equal access to HD for authorized SU via HD access bodies while preserving privacy.^2,103,104^ Consequently, this common framework represents a significant step forward in establishing structured governance for cross-border HD sharing while addressing ethical concerns about data protection and other identified public preferences. Most EU-wide primary studies (17%) were conducted in United Kingdom. The United Kingdom has developed a governance framework that shares many elements with the EHDS approach.^105^ The National Health Service (NHS) strategy and initiatives such as NHS DigiTrials prioritize both accelerating the use of HD for research and increasing patient trust through transparency.^106^ In the United Kingdom, there has been significant public controversy over HD-sharing initiatives for SU, notably the care.data program, which was ultimately abandoned due to public concerns about transparency and consent.^107,108^

Only the two SRs by Hutchings et al. compared several European countries.^67,68^ Notably, different consent preferences were found across European countries.^68,109,110^ Current evidence shows that the Nordic countries can be considered frontrunners in digital health with HD collection and linkage as a source of complete, reliable, and representative health information for SU, and public health policy in each country, which is also used by research institutions and regulatory authorities worldwide.^111,112^

For example, Swedish and Norwegian systems allow patients to view access logs showing who has viewed their medical records and for what purpose,^113,114^ which was identified as a facilitating factor in our overview.^71^ While the adoption rate of the nationwide online PP in Finland already exceeded 89% in 2020, patients’ awareness of the importance of HD sharing in healthcare remains an ongoing concern in other countries.^61,79,80,111,115,116^ For example, one-third of the German population is unaware of the existence of EHRs.^117^ Only about 1.7% of those with statutory health insurance currently use the EHR service^118^; however, one study showed surprising willingness, openness, and trust in using HD.^119^

In contrast, the U.S. health system reflects a more fragmented approach based on a patchwork of regulations, with the Health Insurance Portability and Accountability Act (HIPAA) serving as the primary legislation governing HD privacy.^120,121^ The notice of privacy practices required by HIPAA typically uses complex legal language that many patients find difficult to understand,^122^ which our findings suggest may be a barrier. Crucially, however, there is a significant gap in awareness and transparency for patients, particularly about how their HD is used beyond PU. A recent interview study of 2025 recommend updating HIPAA, including authorization and accountability processes and opt-out options.^123^ Greater transparency could have a threefold positive impact on the acceptance of HD sharing. On the one hand, it could overcome the barrier of concerns about commercial use when the purpose of the SU is research, for example. Widespread concerns about perceived profit motives have a negative impact on HD sharing.^64^ On the other hand, the public benefit of improving medical care could be emphasized, as the benefits to the population may outweigh the risks, with a positive impact on HIE.^71^ Finally, the aspect of social responsibility as a facilitator to share HD could be addressed,^64,67,68,73^ in particular giving something back to other people or future generations showed a positive influence on the willingness to share HD.^73^ In the United States, the “Trusted Exchange Framework and Common Agreement” went live in December 2023 with the aim to improve interoperability and HD sharing through common standards among participating healthcare providers, patients, public health agencies, and payers with strong privacy and security protections.^124^ This initiative could in turn address factors such as data security concerns, improve trust and transparency, and ultimately increase the willingness to share HD. However, significant gaps remain for a seamless nationwide HIE.

The overall quality rating based on R-AMSTAR2 showed that all 11 included SRs were of critically low quality. Therefore, strong doubts exist about the validity and reliability of our findings. Consequently, the derivation of reliable recommendations for practice is severely limited by the available evidence. Our results strongly highlight the need for more high-quality SRs.

### Strengths and limitations

To our knowledge, our work is the first to provide an international theory- and evidence-based overview of the factors influencing patients’ willingness to share their digital HD for PU and SU, independent of study design, HD type, and healthcare technology type, qualitatively assessing the evidence. However, due to the different regulations and HD-sharing practices around the world, some caution is still required when deriving interpretations for a specific country. Separating the factors influencing HD sharing for PU and SU enables readers to focus on findings of interest.

To summarize a maximally comprehensive body of literature, we have used a diverse set of appropriate search terms and followed a sensitive search approach to identify as many relevant SRs as possible. We defined SRs as reviews, applying a comprehensive, reproducible search strategy and critically appraising study quality. However, three included SRs^64,66,70^ included reviews in addition to primary studies, as is not common in SRs. Duplicate articles were excluded to ensure the most unbiased reporting of the results possible.

We categorized the available evidence by accounting for existing technology acceptance and datasharing models to provide a broader understanding of the contribution to digital HD sharing^125^ and address the common criticism that health informatics research is often not grounded in theory.^126–128^ However, seven of the included SRs^64–70^ did not follow a theory-based approach for evidence synthesis, which sometimes made it difficult to compare and synthesize results when conducting meta-synthesis.

We only extracted patients’ views on sharing their digital HD for PU and SU according to our research question, although some SRs also included the opinions of other stakeholders. The literature highlights the relevance of patient involvement. For example, an essential part of the modernization agenda of the UK NHS is providing healthcare responsive to the individual needs of patients, especially those with long-term conditions.^129^ Patient participation is also emphasized at various points in the German Health Data Use Act.^130^ We defined patients as all individuals who have been and/or are in contact with the healthcare system in the past and/or present, encompassing lay persons, the general public, and those who are unwell. Most SRs included a mix of healthy individuals and patients with diverse diseases,^64,67–69,71–73^ which may explain the inconsistent evidence regarding the factor “health status.” Additionally, some information about health status was based on self-reporting, which could also have caused potential bias.

### Implications for future research

This overview includes SRs identified in three major literature databases. Further updated reviews could build on our search and extending it to include additional databases. For certain research questions, such as a country-specific analysis, additional gray literature may be useful to ensure that the most current information is considered, especially given the dynamic nature of digital health. In addition, the views of healthcare professionals (e.g. physicians and nurses) who could influence care and improve health outcomes^131^ and, as described, act as multipliers in involving patients in HD sharing are also particularly relevant for future research. The availability of only low-quality evidence highlights the urgent need for high-quality SRs to provide reliable recommendations for practice. Future research could broaden our evidence map to a holistic framework model to verify which of the 41 factors are especially relevant and to what extent, as well as whether all required variables have been captured. Additionally, our results can be used to develop and test hypotheses about possible correlations between the influencing factors. Further research could also investigate the subsequent effects of HD sharing in clinical or patient-reported outcomes, such as patient satisfaction, behavior, and empowerment. It is also essential to examine the factors influencing the long-term use of healthcare technologies to develop a comprehensive understanding since most existing studies have focused on factors influencing the intention to use or the initial use.

## Conclusion

Our overview provides a theory-based meta-level synthesis of the factors influencing patients’ willingness to share their HD for PU and SU. It identified 41 factors across 15 categories, with 25 consistently identified as facilitators and 11 consistently identified as barriers. It has shown that influencing patients’ willingness to share their HD is a complex challenge. However, policymakers, healthcare providers, and researchers should focus on modifiable factors to increase patients’ willingness to share their HD, such as expected individual usefulness, public benefit, privacy and security concerns, discrimination and stigmatization concerns, trust, confidentiality, and transparency.

Therefore, it is essential to involve future user groups when developing HD-sharing approaches from the outset. Additionally, healthcare professionals play a key role as change agents in patients’ willingness to share their HD. As multipliers, they should be empowered to inform patients about the benefits and risks of HD sharing for PU and SU. In all of these measures, it is also important to transparently communicate the differences between the two HD sharing options: PU and SU.

The low methodological quality of the 11 included SRs emphasizes the need for high-quality SRs to derive reliable, evidence-based recommendations and to develop a holistic framework model for practice.

## Supplemental Material

sj-docx-1-dhj-10.1177_20552076251340254 - Supplemental material for Factors influencing patients’ willingness to share their digital health data for primary and secondary use: A theory- and evidence-based overview of reviews Supplemental material, sj-docx-1-dhj-10.1177_20552076251340254 for Factors influencing patients’ willingness to share their digital health data for primary and secondary use: A theory- and evidence-based overview of reviews by Sabrina Fesl, Caroline Lang, Jochen Schmitt, Stefanie Brückner, Stephen Gilbert, Stefanie Deckert and Madlen Scheibe in DIGITAL HEALTH

sj-xlsx-2-dhj-10.1177_20552076251340254 - Supplemental material for Factors influencing patients’ willingness to share their digital health data for primary and secondary use: A theory- and evidence-based overview of reviews Supplemental material, sj-xlsx-2-dhj-10.1177_20552076251340254 for Factors influencing patients’ willingness to share their digital health data for primary and secondary use: A theory- and evidence-based overview of reviews by Sabrina Fesl, Caroline Lang, Jochen Schmitt, Stefanie Brückner, Stephen Gilbert, Stefanie Deckert and Madlen Scheibe in DIGITAL HEALTH

sj-xlsx-3-dhj-10.1177_20552076251340254 - Supplemental material for Factors influencing patients’ willingness to share their digital health data for primary and secondary use: A theory- and evidence-based overview of reviews Supplemental material, sj-xlsx-3-dhj-10.1177_20552076251340254 for Factors influencing patients’ willingness to share their digital health data for primary and secondary use: A theory- and evidence-based overview of reviews by Sabrina Fesl, Caroline Lang, Jochen Schmitt, Stefanie Brückner, Stephen Gilbert, Stefanie Deckert and Madlen Scheibe in DIGITAL HEALTH

sj-docx-4-dhj-10.1177_20552076251340254 - Supplemental material for Factors influencing patients’ willingness to share their digital health data for primary and secondary use: A theory- and evidence-based overview of reviews Supplemental material, sj-docx-4-dhj-10.1177_20552076251340254 for Factors influencing patients’ willingness to share their digital health data for primary and secondary use: A theory- and evidence-based overview of reviews by Sabrina Fesl, Caroline Lang, Jochen Schmitt, Stefanie Brückner, Stephen Gilbert, Stefanie Deckert and Madlen Scheibe in DIGITAL HEALTH

sj-docx-5-dhj-10.1177_20552076251340254 - Supplemental material for Factors influencing patients’ willingness to share their digital health data for primary and secondary use: A theory- and evidence-based overview of reviews Supplemental material, sj-docx-5-dhj-10.1177_20552076251340254 for Factors influencing patients’ willingness to share their digital health data for primary and secondary use: A theory- and evidence-based overview of reviews by Sabrina Fesl, Caroline Lang, Jochen Schmitt, Stefanie Brückner, Stephen Gilbert, Stefanie Deckert and Madlen Scheibe in DIGITAL HEALTH

sj-xlsx-6-dhj-10.1177_20552076251340254 - Supplemental material for Factors influencing patients’ willingness to share their digital health data for primary and secondary use: A theory- and evidence-based overview of reviews Supplemental material, sj-xlsx-6-dhj-10.1177_20552076251340254 for Factors influencing patients’ willingness to share their digital health data for primary and secondary use: A theory- and evidence-based overview of reviews by Sabrina Fesl, Caroline Lang, Jochen Schmitt, Stefanie Brückner, Stephen Gilbert, Stefanie Deckert and Madlen Scheibe in DIGITAL HEALTH
