# Supplementary material for: Factors influencing patients’ willingness to share their digital health data for primary and secondary use: A theory- and evidence-based overview of reviews
Source: Digit Health. 2025 Jun 30;11:20552076251340254. doi: 10.1177/20552076251340254 (PMC12209586; doi:10.1177/20552076251340254)
Supplement: sj-docx-4-dhj-10.1177_20552076251340254 - Supplemental material for Factors influencing patients’ willingness to share their digital health data for primary and secondary use: A theory- and evidence-based overview of reviews [file sj-docx-4-dhj-10.1177_20552076251340254.docx]

**List of excluded reviews with (multiple) reason(s) for exclusion after screening for eligibility.**

| Reason for exclusion* | References |
| --- | --- |
| Wrong population | **^1, 3, 4, 12, 14, 17, 23, 38, 43, 50, 53^** |
| Wrong concept/construct | ^1,^ **^5,^** ^14,^ **^20, 29, 30, 39, 44, 48, 55^** |
| Wrong context | ^1, 5, 14, 20,^ **^28, 41, 54^** |
| Wrong review type | **^9, 25, 27, 33, 35,^** ^41^**^, 47,^** ^48^**^, 52,^** ^54^**^, 56, 61, 64, 70, 71, 73, 75, 77^** |
| Reports not retrieved | **^8, 18, 22, 34, 46,^** ^48^**^, 57, 69, 79^** |
| Missing critical appraisal | **^2,^** ^3-5^**^, 6, 7,^** ^9^**^, 10, 11, 13, 15, 16,^** ^17^**^, 19,^** ^20^**^, 21,^** ^23^**^, 24,^** ^25^**^, 26,^** ^29, 30^**^, 31, 32,^** ^33, 35^**^, 36, 37,^** ^38^**^,^** ^39^**^, 40,^** ^41^**^, 42,^** ^43, 44^**^, 35,^** ^47, 48^**^, 49,^** ^50^**^, 51,^** ^52-55^**^, 58-60,^** ^61^**^, 62, 63, 65-67,^** ^70^**^, 72,^** ^73^**^, 74,^** ^75^**^, 76,^** ^77^**^, 78^** |
| No peer-reviewed article | ^14, 27, 48,^ **^68^**^, 72, 74, 77, 78^ |

*all applicable exlusion criteria, decisive exclusion criteria in bold

**List of excluded reviews with decisive exclusion criteria according to priorized weighting, as depicted in PRISMA-flowchart.**

| Reason for exclusion | References |
| --- | --- |
| Wrong population (*n* = 11) | ^1, 3, 4, 12, 14, 17, 23, 38, 43, 50, 53^ |
| Wrong concept/construct (*n* = 8) | ^5, 20, 29, 30, 39, 44, 48, 55^ |
| Wrong context (*n* = 3) | ^28, 41, 54^ |
| Wrong review type (*n* = 15) | ^9, 25, 27, 33, 35, 47, 52, 56, 61, 64, 70, 71, 73, 75, 77^ |
| Reports not retrieved (*n* = 8) | ^8, 18, 22, 34, 46, 57, 69, 79^ |
| Missing critical appraisal (*n* = 33) | ^2, 6, 7, 10, 11, 13, 15, 16, 19, 21, 24, 26, 31, 32, 36, 37, 40, 42, 45, 49, 51, 58-60, 62, 63, 65-67, 72, 74, 76, 78^ |
| No peer-reviewed article (*n* = 1) | ^68^ |

1. Anglemyer A, Moore THM, Parker L, et al. Digital contact tracing technologies in epidemics: a rapid review. *Cochrane Database of Systematic Reviews*; 2020: CD013699.

2. Avdagovska M, Menon D and Stafinski T. Capturing the Impact of Patient Portals Based on the Quadruple Aim and Benefits Evaluation Frameworks: Scoping Review. *Journal of medical Internet research* 2020; 22: e24568.

3. Bull S, Cheah PY, Denny S, et al. Best Practices for Ethical Sharing of Individual-Level Health Research Data From Low- and Middle-Income Settings. *Journal of empirical research on human research ethics : JERHRE* 2015; 10: 302-313.

4. Bull S, Roberts N and Parker M. Views of Ethical Best Practices in Sharing Individual-Level Data From Medical and Public Health Research: A Systematic Scoping Review. *Journal of empirical research on human research ethics:JERHRE* 2015; 10: 225-238.

5. Cabieses B, Obach A, Campana C, et al. Revisando Conceptos de Acceso, Trayectorias, Participacion y Conocimiento Tacito en Investigaciones Sobre Pacientes y Cobertura en Salud. *Value in Health Regional Issues* 2023; 33: 42-48.

6. Cardillo L, Cahill F, Wylie H, et al. Patients' perspectives on opt-out consent for observational research: systematic review and focus group. *Br J Nurs* 2018; 27: 1321-1329. 2018/12/12.

7. Coughlin SS, Prochaska JJ, Williams LB, et al. Patient web portals, disease management, and primary prevention. *Risk Manag Healthc Policy* 2017; 10: 33-40. 2017/04/25.

8. Cresswell K and Sheikh A. Organizational issues in the implementation and adoption of health information technology innovations: an interpretative review. *Int J Med Inform* 2013; 82: e73-86. 2012/11/14.

9. Cumyn A, Menard JF, Barton A, et al. Patients' and Members of the Public's Wishes Regarding Transparency in the Context of Secondary Use of Health Data: Scoping Review. *Journal of Medical Internet Research* 2023; 25: e45002.

10. D'Costa SN, Kuhn IL and Fritz Z. A systematic review of patient access to medical records in the acute setting: practicalities, perspectives and ethical consequences. *BMC medical ethics* 2020; 21: 18.

11. De Man Y, Wieland-Jorna Y, Torensma B, et al. Opt-In and Opt-Out Consent Procedures for the Reuse of Routinely Recorded Health Data in Scientific Research and Their Consequences for Consent Rate and Consent Bias: Systematic Review. *Journal of Medical Internet Research* 2023; 25: e42131.

12. De Sutter E, Zace D, Boccia S, et al. Implementation of electronic informed consent in biomedical research and stakeholders' perspectives: Systematic review. *Journal of Medical Internet Research* 2020; 22: e19129.

13. Domaradzki J and Pawlikowski J. Public Attitudes toward Biobanking of Human Biological Material for Research Purposes: A Literature Review. *Int J Environ Res Public Health* 2019; 16 20190621.

14. Dunn M, Strnadova I, Scully JL, et al. Exploring the Barriers and Enablers for the Equitable and Accessible Informed Healthcare Consent Process for People with Intellectual Disability: A Systematic Literature Review. *medRxiv* 2023.

15. Eden KB, Totten AM, Kassakian SZ, et al. Barriers and facilitators to exchanging health information: a systematic review. *Int J Med Inform* 2016; 88: 44-51. 2016/02/18.

16. Esmaeilzadeh P and Sambasivan M. Patients' support for health information exchange: a literature review and classification of key factors. *BMC medical informatics and decision making* 2017; 17: 33.

17. Fennelly O, Cunningham C, Grogan L, et al. Successfully implementing a national electronic health record: a rapid umbrella review. *International Journal of Medical Informatics* 2020; 144. Review.

18. Fisher-Grace K, Turk MT, Anthony MK, et al. Use of Personal Health Records to Support Diabetes Self-management: An Integrative Review. *Comput Inform Nurs* 2021; 39: 298-305. 2020/12/15.

19. Garrison NA, Sathe NA, Antommaria AH, et al. A systematic literature review of individuals' perspectives on broad consent and data sharing in the United States. *Genet Med* 2016; 18: 663-671. 2015/11/20.

20. Genevieve LD, Martani A, Mallet MC, et al. Factors influencing harmonized health data collection, sharing and linkage in Denmark and Switzerland: A systematic review. *PloS one* 2019; 14: e0226015.

21. Gesulga JM, Berjame A, Moquiala KS, et al. Barriers to Electronic Health Record System Implementation and Information Systems Resources: A Structured Review. *Procedia Comput Sci* 2017; 124: 544-551.

22. Goldzweig CL, Orshansky G, Paige NM, et al. Electronic patient portals: evidence on health outcomes, satisfaction, efficiency, and attitudes: a systematic review. *Ann Intern Med* 2013; 159: 677-687. 2013/11/20.

23. Hemsley B, Georgiou A, Carter R, et al. Use of the My Health Record by people with communication disability in Australia: A review to inform the design and direction of future research. *Health inf manag* 2016; 45: 107-115.

24. Hemsley B, Rollo M, Georgiou A, et al. The health literacy demands of electronic personal health records (e-PHRs): An integrative review to inform future inclusive research. *Patient Education and Counseling* 2018; 101: 2-15.

25. Hermansen A, Regier DA and Pollard S. Developing Data Sharing Models for Health Research with Real-World Data: A Scoping Review of Patient and Public Preferences. *J Med Syst* 2022; 46: 86. 2022/10/23.

26. Hill EM, Turner EL, Martin RM, et al. "Let's get the best quality research we can": public awareness and acceptance of consent to use existing data in health research: a systematic review and qualitative study. *BMC medical research methodology* 2013; 13: 72.

27. Hopkinson D. INTEGRATION AND PERCEPTION OF EHEALTH IN THE PRIMARY CARE SETTING. *Value in Health* 2018; 21: S325.

28. Howe N, Giles E, Newbury-Birch D, et al. Systematic review of participants' attitudes towards data sharing: a thematic synthesis. *J Health Serv Res Policy* 2018; 23: 123-133. 2018/04/15.

29. Hubner UH, Egbert N and Schulte G. Clinical Information Systems - Seen through the Ethics Lens. *Yearbook of medical informatics* 2020; 29: 104-114.

30. Husedzinovic A, Ose D, Schickhardt C, et al. Stakeholders' perspectives on biobank-based genomic research: Systematic review of the literature. *European Journal of Human Genetics* 2015; 23: 1607-1614.

31. Irizarry T, DeVito Dabbs A and Curran CR. Patient Portals and Patient Engagement: A State of the Science Review. *J Med Internet Res* 2015; 17: e148. 2015/06/25.

32. Jilka SR, Callahan R, Sevdalis N, et al. "Nothing About Me Without Me": An Interpretative Review of Patient Accessible Electronic Health Records. *Journal of medical Internet research* 2015; 17: e161.

33. Kalkman S, van Delden J, Banerjee A, et al. Patients' and public views and attitudes towards the sharing of health data for research: a narrative review of the empirical evidence. *J Med Ethics* 2022; 48: 3-13. 2019/11/14.

34. Kallmerten PS, Chia LR, Jakub K, et al. Patient Portal Use by Adults With Heart Failure: An Integrative Review. *Computers, informatics, nursing:CIN* 2021; 39: 418-431.

35. Kariotis TC, Prictor M, Chang S, et al. Impact of Electronic Health Records on Information Practices in Mental Health Contexts: Scoping Review. *Journal of Medical Internet Research* 2022; 24: e30405.

36. Kassam I, Ilkina D, Kemp J, et al. Patient Perspectives and Preferences for Consent in the Digital Health Context: State-of-the-art Literature Review. *J Med Internet Res* 2023; 25: e42507.

37. Kelly MM, Coller RJ and Hoonakker PL. Inpatient Portals for Hospitalized Patients and Caregivers: A Systematic Review. *Journal of hospital medicine* 2018; 13: 405-412.

38. Kloss LL, Brodnik MS and Rinehart-Thompson LA. Access and Disclosure of Personal Health Information: A Challenging Privacy Landscape in 2016-2018. *Yearbook of medical informatics* 2018; 27: 60-66.

39. Kneale L and Demiris G. Lack of Diversity in Personal Health Record Evaluations with Older Adult Participants: A Systematic Review of Literature. *J Innov Health Inform* 2017; 23: 881. 2017/03/28.

40. Kruse CS, Argueta DA, Lopez L, et al. Patient and provider attitudes toward the use of patient portals for the management of chronic disease: a systematic review. *J Med Internet Res* 2015; 17: e40. 2015/02/24.

41. Lang M, Lemieux S, Hebert J, et al. Legal and ethical considerations for the design and use of web portals for researchers, clinicians, and patients: Scoping literature review. *Journal of Medical Internet Research* 2021; 23: e26450.

42. Lea NC, Nicholls J and Fitzpatrick NK. Between Scylla and Charybdis: Charting the Wicked Problem of Reusing Health Data for Clinical Research Informatics. *Yearbook of medical informatics* 2018; 27: 170-176.

43. Li X and Cong Y. A Systematic Literature Review of Ethical Challenges Related to Medical and Public Health Data Sharing in China. *Journal of empirical research on human research ethics:JERHRE* 2021; 16: 537-554.

44. Liu J, Luo L, Zhang R, et al. Patient satisfaction with electronic medical/health record: A systematic review. *Scandinavian Journal of Caring Sciences* 2013; 27: 785-791.

45. Naeem I, Quan H, Singh S, et al. Factors Associated With Willingness to Share Health Information: Rapid Review. *JMIR Human Factors* 2022; 9:1.

46. Nguyen L, Bellucci E and Nguyen LT. Electronic health records implementation: an evaluation of information system impact and contingency factors. *Int J Med Inform* 2014; 83: 779-796. 2014/08/03.

47. Ose D, Baudendistel I, Pohlmann S, et al. Personal health records on the Internet. A narrative review of attitudes, expectations, utilization and effects on health outcomes. *Zeitschrift fur Evidenz, Fortbildung und Qualitat im Gesundheitswesen* 2017; 122: 9-21.

48. Osovskaya I. Personal health records: Understanding the factors that contribute to creating value and practical use by patients and citizens. In: *Proceedings of the 2017 International Conference on Digital Health,* London, United Kingdom (DH '17), pp.237-238. New York: Association for Computing Machinery.

49. Otte-Trojel T, de Bont A, Rundall TG, et al. What do we know about developing patient portals? A systematic literature review. *J Am Med Inform Assoc* 2016; 23: e162-168. 2015/09/04.

50. Piasecki J, Walkiewicz-Zarek E, Figas-Skrzypulec J, et al. Ethical issues in biomedical research using electronic health records: a systematic review. *Medicine, health care, and philosophy* 2021; 24: 633-658.

51. Powell KR. Patient-Perceived Facilitators of and Barriers to Electronic Portal Use: A Systematic Review. *Computers, informatics, nursing:CIN* 2017; 35: 565-573.

52. Raspa M, Moultrie R, Wagner L, et al. Ethical, legal, and social issues related to the inclusion of individuals with intellectual disabilities in electronic health record research: Scoping review. *Journal of Medical Internet Research* 2020; 22: e16734.

53. Ratwani R, Fairbanks T, Savage E, et al. Mind the Gap. A systematic review to identify usability and safety challenges and practices during electronic health record implementation. *Applied clinical informatics* 2016; 7: 1069-1087.

54. Rempel ES, Barnett J and Durrant H. Public engagement with UK government data science: Propositions from a literature review of public engagement on new technologies. *Government Information Quarterly* 2018; 35: 569-578.

55. Roehrs A, da Costa CA, Righi RD, et al. Personal Health Records: A Systematic Literature Review. *Journal of medical Internet research* 2017; 19: e13.

56. Ross J, Stevenson F, Lau R, et al. Factors that influence the implementation of e-health: a systematic review of systematic reviews (an update). *Implementation science:IS* 2016; 11: 146.

57. Rudin RS, Motala A, Goldzweig CL, et al. Usage and effect of health information exchange: A systematic review. *Annals of Internal Medicine* 2014; 161: 803-811.

58. Sakaguchi-Tang DK, Bosold AL, Choi YK, et al. Patient Portal Use and Experience Among Older Adults: Systematic Review. *JMIR Med Inform* 2017; 5: e38. 2017/10/19.

59. Sartain SA, Stressing S and Prieto J. Patients' views on the effectiveness of patient-held records: a systematic review and thematic synthesis of qualitative studies. *Health expect* 2015; 18: 2666-2677.

60. Shabani M, Bezuidenhout L and Borry P. Attitudes of research participants and the general public towards genomic data sharing: a systematic literature review. *Expert Rev Mol Diagn* 2014; 14: 1053-1065. 20140926.

61. Shah SD and Liebovitz D. It Takes Two to Tango: Engaging Patients and Providers With Portals. *PM and R* 2017; 9: S85-S97.

62. Shen N, Bernier T, Sequeira L, et al. Understanding the patient privacy perspective on health information exchange: A systematic review. *Int J Med Inform* 2019; 125: 1-12. 2019/03/28.

63. Showell C. Barriers to the use of personal health records by patients: a structured review. *PeerJ* 2017; 5: e3268. 2017/05/04.

64. Simpson E, Brown R, Sillence E, et al. Understanding the Barriers and Facilitators to Sharing Patient-Generated Health Data Using Digital Technology for People Living With Long-Term Health Conditions: A Narrative Review. *Front Public Health* 2021; 9: 641424. 2021/12/11.

65. Skovgaard LL, Wadmann S and Hoeyer K. A review of attitudes towards the reuse of health data among people in the European Union: The primacy of purpose and the common good. *Health Policy* 2019; 123: 564-571. 2019/04/10.

66. Soni H, Grando A, Murcko A, et al. State of the art and a mixed-method personalized approach to assess patient perceptions on medical record sharing and sensitivity. *Journal of Biomedical Informatics* 2020; 101: 103338.

67. Staccini P and Lau AYS. Findings from 2017 on Consumer Health Informatics and Education: Health Data Access and Sharing. *Yearbook of medical informatics* 2018; 27: 163-169.

68. Street J, Carter S, Fabrianesi B, et al. "Public attitudes to sharing government data with private industry: a systematic scoping review". *medRxiv* 2022.

69. Thompson MJ, Reilly JD and Valdez RS. Work system barriers to patient, provider, and caregiver use of personal health records: A systematic review. *Appl Ergon* 2016; 54: 218-242.

70. Van Kasteren Y, Maeder A, Williams PA, et al. Consumer Perspectives on MyHealth Record: A Review. *Stud Health Technol Inform* 2017; 239: 146-152. 2017/08/02.

71. Van Mens HJT, Duijm RD, Nienhuis R, et al. Determinants and outcomes of patient access to medical records: Systematic review of systematic reviews. *Int J Med Inform* 2019; 129: 226-233.

72. Van Panhuis WG, Paul P, Emerson C, et al. A systematic review of barriers to data sharing in public health. *BMC Public Health* 2014; 14: 1144. 2014/11/08.

73. Waind E. Trust, security and public interest: striking the balance A narrative review of previous literature on public attitudes towards the sharing, linking and use of administrative data for research. *Int J Popul Data Sci* 2020; 5: 1368. 2021/05/27.

74. Weber AS, Turjoman R, Shaheen Y, et al. Systematic thematic review of e-health research in the Gulf Cooperation Council (Arabian Gulf): Bahrain, Kuwait, Oman, Qatar, Saudi Arabia and United Arab Emirates. *Journal of telemedicine and telecare* 2017; 23: 452-459.

75. Woodbury RB, Beans JA, Hiratsuka VY, et al. Data Management in Health-Related Research Involving Indigenous Communities in the United States and Canada: A Scoping Review. *Frontiers in Genetics* 2019; 10: 942.

76. Wu H and LaRue EM. Linking the health data system in the U.S.: Challenges to the benefits. *International Journal of Nursing Sciences* 2017; 4: 410-417.

77. Zadeh PE and Maddah M. The role of consumer consent in Health Information Exchange (HIE). In: *AMCIS 2017 - 23rd Americas Conference on Information Systems: A Tradition of Innovation,* Boston, USA 2017.

78. Zhang Y, Fleischmann KR, Gao J, et al. A systematic review of the literature on consumers' use of patient portals: Preliminary results. *PROCEEDINGS OF THE ASSOCIATION FOR INFORMATION SCIENCE AND TECHNOLOGY* 2016; 52: 1-4.

79. Zhang T, Shen N, Booth R, et al. Supporting the use of patient portals in mental health settings: a scoping review. *Inform Health Soc Care* 2022; 47: 62-79. 2021/05/26.
