# Supplementary material for: Factors influencing patients’ willingness to share their digital health data for primary and secondary use: A theory- and evidence-based overview of reviews
Source: Digit Health. 2025 Jun 30;11:20552076251340254. doi: 10.1177/20552076251340254 (PMC12209586; doi:10.1177/20552076251340254)
Supplement: sj-docx-5-dhj-10.1177_20552076251340254 - Supplemental material for Factors influencing patients’ willingness to share their digital health data for primary and secondary use: A theory- and evidence-based overview of reviews [file sj-docx-5-dhj-10.1177_20552076251340254.docx]

| Duplicate reference | No. of duplication(s) | Related reviews of duplication(s) |
| --- | --- | --- |
| ^1^ | 2 | - Hutchings et al. (2020) - Hutchings et al. (2021) - Stockdale et al. (2019) |
| ^2^ | 2 | - Hutchings et al. (2020) - Hutchings et al. (2021) - Stockdale et al. (2019) |
| ^3^ | 1 | - Amante et al. (2014) - Mold et al. (2018) |
| ^4^ | 1 | - Hutchings et al. (2020) - Hutchings et al. (2021) |
| ^5^ | 2 | - Hutchings et al. (2020) - Hutchings et al. (2021) - Stockdale et al. (2019) |
| ^6^ | 1 | - Hutchings et al. (2020) - Hutchings et al. (2021) |
| ^7^ | 2 | - Aitken et al. (2016) - Hutchings et al. (2020) - Hutchings et al. (2021) |
| ^8^ | 1 | - Hutchings et al. (2020) - Hutchings et al. (2021) |
| ^9^ | 1 | - Hutchings et al. (2020) - Hutchings et al. (2021) |
| ^10^ | 1 | - Aitken et al. (2016) - Dendere et al. (2019) |
| ^11^ | 1 | - Abd-Alrazaq et al. (2019) - Dendere et al. (2019) |
| ^12^ | 3 | - Aitken et al. (2016) - Hutchings et al. (2020) - Hutchings et al. (2021) |
| ^13^ | 1 | - Amante et al. (2014) - Mold et al. (2018) |
| ^14^ | 1 | - Hutchings et al. (2020) - Hutchings et al. (2021) |
| ^15^ | 2 | - Abd-Alrazaq et al. (2019) - Amante et al. (2014) - Mold et al. (2018) |
| ^16^ | 2 | - Aitken et al. (2016) - Hutchings et al. (2021) - Stockdale et al. (2019) |
| ^17^* | 2 | - Hutchings et al. (2020) - Hutchings et al. (2021) - Stockdale et al. (2019) |
| ^18^* | 2 | - Hutchings et al. (2020) - Hutchings et al. (2021) - Stockdale et al. (2019) |
| ^19^ | 1 | - Hutchings et al. (2020) - Hutchings et al. (2021) |
| ^20^ | 1 | - Hutchings et al. (2020) - Hutchings et al. (2021) |
| ^21^ | 2 | - Abd-Alrazaq et al. (2019) - Amante et al. (2014) - Mold et al. (2018) |
| ^22^ | 1 | - Abd-Alrazaq et al. (2019) - Amante et al. (2014) |
| ^23^ | 1 | - Abd-Alrazaq et al. (2019) - Amante et al. (2014) |
| ^24^ | 1 | - Abd-Alrazaq et al. (2019) - Dendere et al. (2019) |
| ^25^ | 1 | - Hutchings et al. (2020) - Hutchings et al. (2021) |
| ^26^ | 1 | - Abd-Alrazaq et al. (2019) - Amante et al. (2014) |
| ^27^ | 1 | - Hutchings et al. (2020) - Hutchings et al. (2021) |
| ^28^ | 1 | - Abd-Alrazaq et al. (2019) - Mold et al. (2015) |
| ^29^ | 1 | - Hutchings et al. (2020) - Hutchings et al. (2021) |
| ^30^ | 1 | - Abd-Alrazaq et al. (2019) - Moon et al. (2017) |
| ^31^ | 1 | - Abd-Alrazaq et al. (2019) - Moon et al. (2017) |
| ^32^ | 1 | - Abd-Alrazaq et al. (2019) - Moon et al. (2017) |
| ^33^ | 1 | - Abd-Alrazaq et al. (2019) - Dendere et al. (2019) |
| ^34^* | 1 | - Hutchings et al. (2020) - Hutchings et al. (2021) |
| ^35^ | 1 | - Abd-Alrazaq et al. (2019) - Mold et al. (2018) |
| ^36^ | 1 | - Abd-Alrazaq et al. (2019) - Mold et al. (2018) |
| ^37^ | 1 | - Abd-Alrazaq et al. (2019) - Mold et al. (2018) |
| ^38^ | 1 | - Abd-Alrazaq et al. (2019) - Mold et al. (2018) |
| ^39^ | 1 | - Hutchings et al. (2020) - Hutchings et al. (2021) |
| ^40^ | 2 | - Abd-Alrazaq et al. (2019) - Amante et al. (2014) - Mold et al. (2018) |
| ^41^ | 2 | - Abd-Alrazaq et al. (2019) - Amante et al. (2014) - Mold et al. (2018) |
| ^42^ | 1 | - Hutchings et al. (2020) - Hutchings et al. (2021) |
| ^43^ | 2 | - Abd-Alrazaq et al. (2019) - Amante et al. (2014) - Mold et al. (2018) |
| ^44^ | 1 | - Hutchings et al. (2020) - Hutchings et al. (2021) |
| ^45^ | 1 | - Amante et al. (2014) - Mold et al. (2018) |
| ^46^ | 2 | - Abd-Alrazaq et al. (2019) - Amante et al. (2014) - Mold et al. (2018) |
| ^47^ | 1 | - Hutchings et al. (2020) - Hutchings et al. (2021) |
| ^48^ | 1 | - Hutchings et al. (2020) - Hutchings et al. (2021) |
| ^49^ | 1 | - Abd-Alrazaq et al. (2019) - Dendere et al. (2019) |
| ^50^ | 1 | - Hutchings et al. (2020) - Hutchings et al. (2021) |
| ^51^ | 1 | - Abd-Alrazaq et al. (2019) - Amante et al. (2014) |

* gray literature

1. Audrey S, Brown L, Campbell R, et al. Young people's views about consenting to data linkage: findings from the PEARL qualitative study. *BMC Med Res Methodol* 2016; 16: 34. 2016/03/24.

2. Barrett G, Cassell JA, Peacock JL, et al. National survey of British public's views on use of identifiable medical data by the National Cancer Registry. *Bmj* 2006; 332: 1068-1072. 2006/05/02.

3. Bredfeldt CE, Compton-Phillips AL and Snyder MH. Effects of between visit physician-patient communication on Diabetes Recognition Program scores. *Int J Qual Health Care* 2011; 23: 664-673. 2011/09/23.

4. Broes S, Verbaanderd C, Casteels M, et al. Sharing of Clinical Trial Data and Samples: The Cancer Patient Perspective. *Front Med (Lausanne)* 2020; 7: 33. 2020/03/03.

5. Campbell B, Thomson H, Slater J, et al. Extracting information from hospital records: what patients think about consent. *Qual Saf Health Care* 2007; 16: 404-408. 2007/12/07.

6. Courbier S, Dimond R and Bros-Facer V. Share and protect our health data: An evidence based approach to rare disease patients' perspectives on data sharing and data protection - Quantitative survey and recommendations. *Orphanet Journal of Rare Diseases* 2019; 14: 175.

7. Damschroder LJ, Pritts JL, Neblo MA, et al. Patients, privacy and trust: patients' willingness to allow researchers to access their medical records. *Soc Sci Med* 2007; 64: 223-235. 2006/10/19.

8. Darquy S, Moutel G, Lapointe AS, et al. Patient/family views on data sharing in rare diseases: study in the European LeukoTreat project. *Eur J Hum Genet* 2016; 24: 338-343. 2015/06/18.

9. Eloranta K and Auvinen A. Population attitudes towards research use of health care registries: a population-based survey in Finland. *BMC Med Ethics* 2015; 16: 48. 2015/07/18.

10. Grant A, Ure J, Nicolson DJ, et al. Acceptability and perceived barriers and facilitators to creating a national research register to enable 'direct to patient' enrolment into research: the Scottish Health Research Register (SHARE). *BMC Health Serv Res* 2013; 13: 422. 2013/10/22.

11. Griffin A, Skinner A, Thornhill J, et al. Patient Portals: Who uses them? What features do they use? And do they reduce hospital readmissions? *Appl Clin Inform* 2016; 7: 489-501. 2016/07/21.

12. Haddow G, Bruce A, Sathanandam S, et al. 'Nothing is really safe': a focus group study on the processes of anonymizing and sharing of health data for research purposes. *J Eval Clin Pract* 2011; 17: 1140-1146. 2010/07/16.

13. Harris LT, Haneuse SJ, Martin DP, et al. Diabetes quality of care and outpatient utilization associated with electronic patient-provider messaging: a cross-sectional analysis. *Diabetes Care* 2009; 32: 1182-1187. 2009/04/16.

14. Hay AE, Leung YW, Pater JL, et al. Linkage of Clinical Trial and Administrative Data: A Survey of Cancer Patient Preferences. *Current Oncology* 2017; 24: 161-167.

15. Hess R, Bryce CL, Paone S, et al. Exploring challenges and potentials of personal health records in diabetes self-management: implementation and initial assessment. *Telemed J E Health* 2007; 13: 509-517. 2007/11/15.

16. Hill EM, Turner EL, Martin RM, et al. "Let's get the best quality research we can": public awareness and acceptance of consent to use existing data in health research: a systematic review and qualitative study. *BMC medical research methodology* 2013; 13: 72.

17. Ipsos MORI Social Research Institute. *Perceptions of the cancer registry: attitudes towards and awareness of cancer data collection.* Report for Macmillan Cancer Support and Cancer Research UK. UK, September 2016.

18. Ipsos MORI Social Research Institute. *The one-way-mirror: Public attitudes to commercial access to health data*. Report prepared for the Wellcome Trust. UK, March 2016.

19. King T, Brankovic L and Gillard P. Perspectives of Australian adults about protecting the privacy of their health information in statistical databases. *Int J Med Inform* 2012; 81: 279-289. 2012/02/07.

20. Lucero RJ, Kearney J, Cortes Y, et al. Benefits and Risks in Secondary Use of Digitized Clinical Data: Views of Community Members Living in a Predominantly Ethnic Minority Urban Neighborhood. *AJOB Empir Bioeth* 2015; 6: 12-22. 2015/06/24.

21. Lyles CR, Harris LT, Jordan L, et al. Patient race/ethnicity and shared medical record use among diabetes patients. *Med Care* 2012; 50: 434-440. 2012/02/23.

22. Lyles CR, Sarkar U, Ralston JD, et al. Patient-provider communication and trust in relation to use of an online patient portal among diabetes patients: The Diabetes and Aging Study. *J Am Med Inform Assoc* 2013; 20: 1128-1131. 2013/05/17.

23. Mayberry LS, Kripalani S, Rothman RL, et al. Bridging the digital divide in diabetes: family support and implications for health literacy. *Diabetes Technol Ther* 2011; 13: 1005-1012. 2011/07/02.

24. Mikles SP and Mielenz TJ. Characteristics of electronic patient-provider messaging system utilisation in an urban health care organisation. *J Innov Health Inform* 2014; 22: 214-221. 2014/01/01.

25. Ni MY, Li TK, Hui RWH, et al. Requesting a unique personal identifier or providing a souvenir incentive did not affect overall consent to health record linkage: evidence from an RCT nested within a cohort. *J Clin Epidemiol* 2017; 84: 142-149. 2017/01/25.

26. Osborn CY, Mayberry LS, Wallston KA, et al. Understanding patient portal use: implications for medication management. *J Med Internet Res* 2013; 15: e133. 2013/07/05.

27. Page SA and Mitchell I. Patients' opinions on privacy, consent and the disclosure of health information for medical research. *Chronic Dis Can* 2006; 27: 60-67.

28. Palen TE, Ross C, Powers JD, et al. Association of online patient access to clinicians and medical records with use of clinical services. *Jama* 2012; 308: 2012-2019. 2012/11/22.

29. Parkin L and Paul C. Public good, personal privacy: a citizens' deliberation about using medical information for pharmacoepidemiological research. *J Epidemiol Community Health* 2011; 65: 150-156. 2009/12/02.

30. Patel VN, Abramson E, Edwards AM, et al. Consumer attitudes toward personal health records in a beacon community. *Am J Manag Care* 2011; 17: e104-120. 2011/07/21.

31. Patel VN, Dhopeshwarkar RV, Edwards A, et al. Low-income, ethnically diverse consumers' perspective on health information exchange and personal health records. *Informatics for Health and Social Care* 2011; 36: 233-252.

32. Patel VN, Dhopeshwarkar RV, Edwards A, et al. Consumer Support for Health Information Exchange and Personal Health Records: A Regional Health Information Organization Survey. *Journal of Medical Systems* 2012; 36: 1043-1052.

33. Ralston JD, Silverberg MJ, Grothaus L, et al. Use of web-based shared medical records among patients with HIV. *Am J Manag Care* 2013; 19: e114-124. 2013/06/04.

34. Robinson, G, Dolk, H, Given, J, et al. Knowledge Exchange Seminar Series 2017-18. Public attitudes to data sharing in Northern Ireland: Findings from the Northern Ireland Life and Times Survey 2015. Ulster: Administratrive Data Research Network; Public Health Agency, Ulster University, 2018.

35. Roblin DW, Houston TK, 2nd, Allison JJ, et al. Disparities in use of a personal health record in a managed care organization. *J Am Med Inform Assoc* 2009; 16: 683-689. 2009/07/02.

36. Ronda MC, Dijkhorst-Oei LT, Gorter KJ, et al. Differences between diabetes patients who are interested or not in the use of a patient Web portal. *Diabetes Technol Ther* 2013; 15: 556-563. 2013/06/20.

37. Ronda MC, Dijkhorst-Oei LT and Rutten GE. Reasons and barriers for using a patient portal: survey among patients with diabetes mellitus. *J Med Internet Res* 2014; 16: e263. 2014/11/27.

38. Ronda MC, Dijkhorst-Oei LT and Rutten GE. Patients' Experiences with and Attitudes towards a Diabetes Patient Web Portal. *PLoS One* 2015; 10: e0129403. 2015/06/19.

39. Sakshaug JW, Couper MP, Ofstedal MB, et al. LINKING SURVEY AND ADMINISTRATIVE RECORDS: MECHANISMS OF CONSENT. *Sociol Methods Res* 2012; 41: 535-569. 2012/11/01.

40. Sarkar U, Karter AJ, Liu JY, et al. The literacy divide: health literacy and the use of an internet-based patient portal in an integrated health system-results from the diabetes study of northern California (DISTANCE). *J Health Commun* 2010; 15 Suppl 2: 183-196. 2010/09/29.

41. Sarkar U, Karter AJ, Liu JY, et al. Social disparities in internet patient portal use in diabetes: evidence that the digital divide extends beyond access. *J Am Med Inform Assoc* 2011; 18: 318-321. 2011/01/26.

42. Slegers C, Zion D, Glass D, et al. Why do people participate in epidemiological research? *J Bioeth Inq* 2015; 12: 227-237. 2015/02/13.

43. Tenforde M, Nowacki A, Jain A, et al. The association between personal health record use and diabetes quality measures. *J Gen Intern Med* 2012; 27: 420-424. 2011/10/19.

44. Tully MP, Bozentko K, Clement S, et al. Investigating the Extent to Which Patients Should Control Access to Patient Records for Research: A Deliberative Process Using Citizens' Juries. *J Med Internet Res* 2018; 20: e112. 2018/03/30.

45. Wade-Vuturo AE, Mayberry LS and Osborn CY. Secure messaging and diabetes management: experiences and perspectives of patient portal users. *J Am Med Inform Assoc* 2013; 20: 519-525. 2012/12/18.

46. Weppner WG, Ralston JD, Koepsell TD, et al. Use of a shared medical record with secure messaging by older patients with diabetes. *Diabetes Care* 2010; 33: 2314-2319. 2010/08/27.

47. Whiddett R, Hunter I, Engelbrecht J, et al. Patients' attitudes towards sharing their health information. *Int J Med Inform* 2006; 75: 530-541. 2005/10/04.

48. Willison DJ, Schwartz L, Abelson J, et al. Alternatives to project-specific consent for access to personal information for health research: what is the opinion of the Canadian public? *J Am Med Inform Assoc* 2007; 14: 706-712. 2007/08/23.

49. Winkelman WJ, Leonard KJ and Rossos PG. Patient-perceived usefulness of online electronic medical records: employing grounded theory in the development of information and communication technologies for use by patients living with chronic illness. *J Am Med Inform Assoc* 2005; 12: 306-314. 2005/02/03.

50. Xafis V. The acceptability of conducting data linkage research without obtaining consent: lay people's views and justifications. *BMC medical ethics* 2015; 16: 79.

51. Zickmund SL, Hess R, Bryce CL, et al. Interest in the use of computerized patient portals: role of the provider-patient relationship. *J Gen Intern Med* 2008; 23 Suppl 1: 20-26. 2008/01/10.
